# Noninvasive prenatal diagnosis targeting fetal nucleated red blood cells

**DOI:** 10.1186/s12951-022-01749-3

**Published:** 2022-12-30

**Authors:** Yanyu Chen, Zhuhao Wu, Joseph Sutlive, Ke Wu, Lu Mao, Jiabao Nie, Xing-Zhong Zhao, Feng Guo, Zi Chen, Qinqin Huang

**Affiliations:** 1 Academy of Medical Sciences, The Second Affiliated Hospital of Zhengzhou University, Zhengzhou University, Zhengzhou, 450052 China; 2 School of Physics and Technology, Wuhan University, Wuhan, 430072 China; 3 Department of Intelligent Systems Engineering, Indiana University, Bloomington, IN 47405 USA; 4 Division of Thoracic and Cardiac Surgery, Brigham and Women’s Hospital and Harvard Medical School, Boston, MA 02115 USA; 5 Department of Biological Sciences, Northeastern University, Boston, MA 02115 USA

**Keywords:** Microfluidics, Microtechnology, Biosensing, Noninvasive prenatal diagnosis, Fetal-nucleated red blood cells

## Abstract

Noninvasive prenatal diagnosis (NIPD) aims to detect fetal-related genetic disorders before birth by detecting markers in the peripheral blood of pregnant women, holding the potential in reducing the risk of fetal birth defects. Fetal-nucleated red blood cells (fNRBCs) can be used as biomarkers for NIPD, given their remarkable nature of carrying the entire genetic information of the fetus. Here, we review recent advances in NIPD technologies based on the isolation and analysis of fNRBCs. Conventional cell separation methods rely primarily on physical properties and surface antigens of fNRBCs, such as density gradient centrifugation, fluorescence-activated cell sorting, and magnetic-activated cell sorting. Due to the limitations of sensitivity and purity in Conventional methods, separation techniques based on micro-/nanomaterials have been developed as novel methods for isolating and enriching fNRBCs. We also discuss emerging methods based on microfluidic chips and nanostructured substrates for static and dynamic isolation of fNRBCs. Additionally, we introduce the identification techniques of fNRBCs and address the potential clinical diagnostic values of fNRBCs. Finally, we highlight the challenges and the future directions of fNRBCs as treatment guidelines in NIPD.

**Graphical Abstract:**

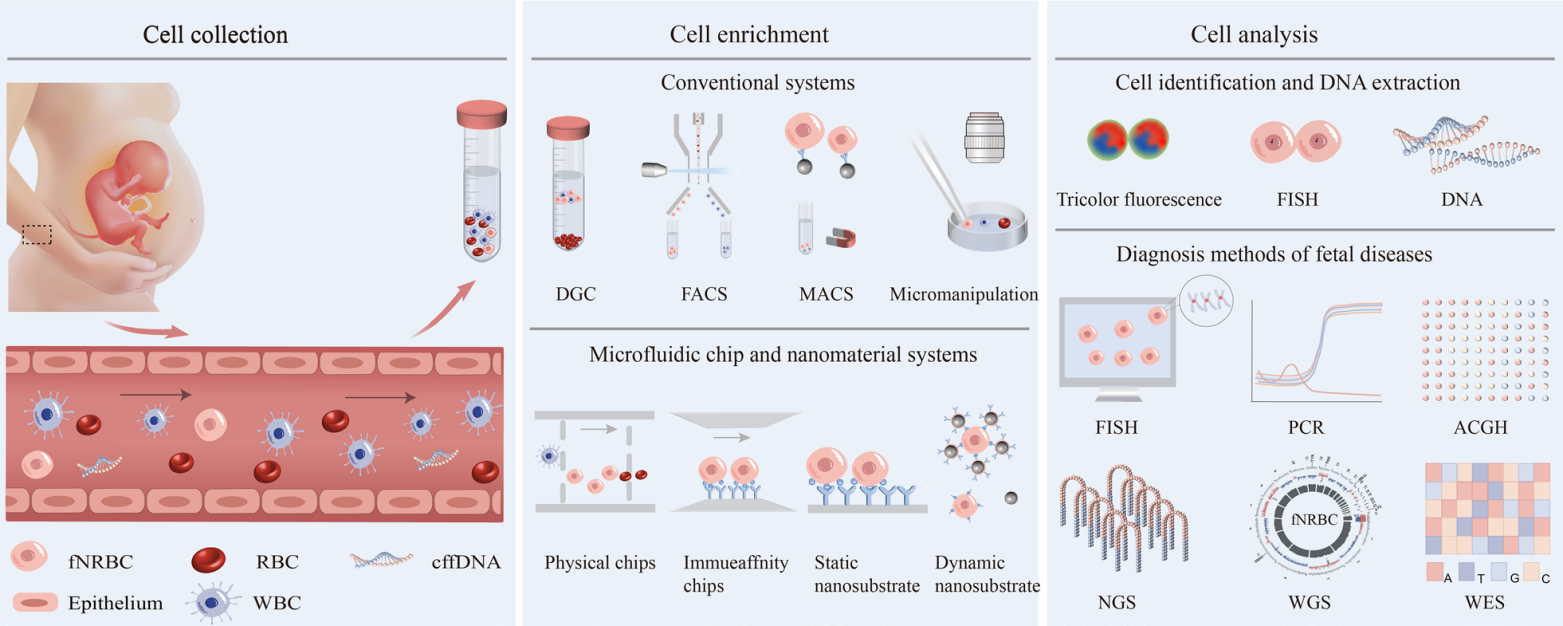

## Introduction

Approximately 8 million infants born in the world every year are diagnosed with fetal genetic disorder, and over 300,000 die from the disease [1]. Fetal genetic disorders including gene mutations and chromosomal abnormalities have been considered as the leading causes of infant mortality. Even if infants with one of these disorders do survive, most of them show intellectual or physical disabilities that cannot be cured. This imposes a serious economic burden on society, as the total hospital cost of treating these diseases exceeds $14 billion in the US [2, 3]. Tremendous techniques have been used in prenatal diagnosis to reduce the risk of fetal birth defects [4]. As shown in Table 1, Conventional techniques, including amniocentesis, fetal umbilical vein puncture, and chorionic villi sampling [5], are used as the gold standard for prenatal diagnosis by far. However, these invasive diagnostic techniques could bring risks such as a miscarriage to the pregnant woman [6]. As an alternative, noninvasive prenatal diagnosis (NIPD) provides a fast, safe, and convenient method for diagnosing fetal diseases in the clinic [4, 7]. Currently, ultrasound and serological tests are also widely used for noninvasive screening but are suffering from low sensitivity and/or low detection, further requiring an invasive gold standard for confirmation [8, 9]. Therefore, there is a critical need to identify more representative markers and relevant techniques in NIPD.

**Table 1:** Strengths and Weaknesses of Prenatal Diagnosis Methods

Methods	Strengths	Weaknesses	References
Invasive	Wide range of clinical applications	Invasive and risky	[5, 6]
Amniocentesis	Gold standard;	Invasive means;	[5]
	For detection of fetal chromosomal abnormalities	Risk of abortion for pregnant women	
Chorionic villi	For karyotyping and genetic diagnosis;	Invasive, with risk of preterm delivery, intracavitary infection	[6]
Sampling	Detectable at 6–9 weeks	and miscarriage;	
		Contaminated samples seriously affect the test accuracy	
Non-invasive	Quick, non-invasive, and convenient	Less clinical application at present	[4, 7]
Serum Testing	Non-invasive, detectable in early pregnancy;	Low sensitivity and specificity;	[8]
	For Down syndrome and neurotuberculosis screening	Only as an aid, need to have invasive methods to confirm	
Ultrasound	Non-invasive, Detect thickness of the nuchal translucency	A complementary tool, more limited in detecting fetal abnormalities	[9]
	to rule out chromosomal abnormalities		
CffDNA	Non-invasive and can be detected as early as 4 weeks;	The minimal, mosaic phenomenon, challenge to detect;	[14–17]
	Contains fetal genetic information for fetal aneuploidy screening	Requires invasive means for confirmation	
FNRBCs	Contain the whole genetic information of the fetus;	Low quantity	[23, 25–27]
	Have specific biomarkers (CD71、CD147、GPA);		
	A short life cycle, and not affected by the last prenatal examination;		
	Can be detected at 6 weeks of gestation		

Current fetal genetic biomarkers in NIPD are mainly derived from cell-free fetal DNA (cffDNA) and fNRBCs in the peripheral blood of pregnant women [10–13]. Since the discovery of cffDNA in the pregnancy peripheral blood in 1997, numerous studies have illustrated the great contribution of cffDNA in fetal aneuploidy screening [14–17]. However, cffDNA is not only minimal in the first trimester but also could contain a mixture of fetal DNA with a large amount of maternal DNA in the placental mosaic. Consequently, it significantly limits the isolation of cffDNA and results in false positives or false negatives, bringing difficulties for downstream analysis in NIPD [18–21]. Superior to cffDNA, fNRBCs may provide a comprehensive and precise result for NIPD. In 1893, Schmorl et al. discovered fNRBCs in maternal pathological autopsies [22]. Later in 1993, Simpson et al. obtained fNRBCs based on markers like transferrin receptor (CD71) and Glycophorin A (GPA) and demonstrated the existence of fetal DNA in maternal blood using PCR [23]. They then detected trisomy 21 (T21) with trisomy 18 (T18) by fluorescence in situ hybridization (FISH), offering the possibility of using fNRBCs for NIPD. FNRBCs are promising biomarkers for NIPD [12, 13, 24] due to several advantages: (1) fNRBCs contain the whole genetic information of the fetus [25]; (2) fNRBCs have distinct biomarkers on their surface to facilitate cell isolation and enrichment, such as CD71, CD147, GPA [26]; (3) The short life cycles of fNRBCs are not affected by the last prenatal examination [27]; (4) fNRBCs can be detected at 6 weeks of gestation and their amounts are positively correlated with gestational weeks in the second trimester [27].

**Fig. 1:**
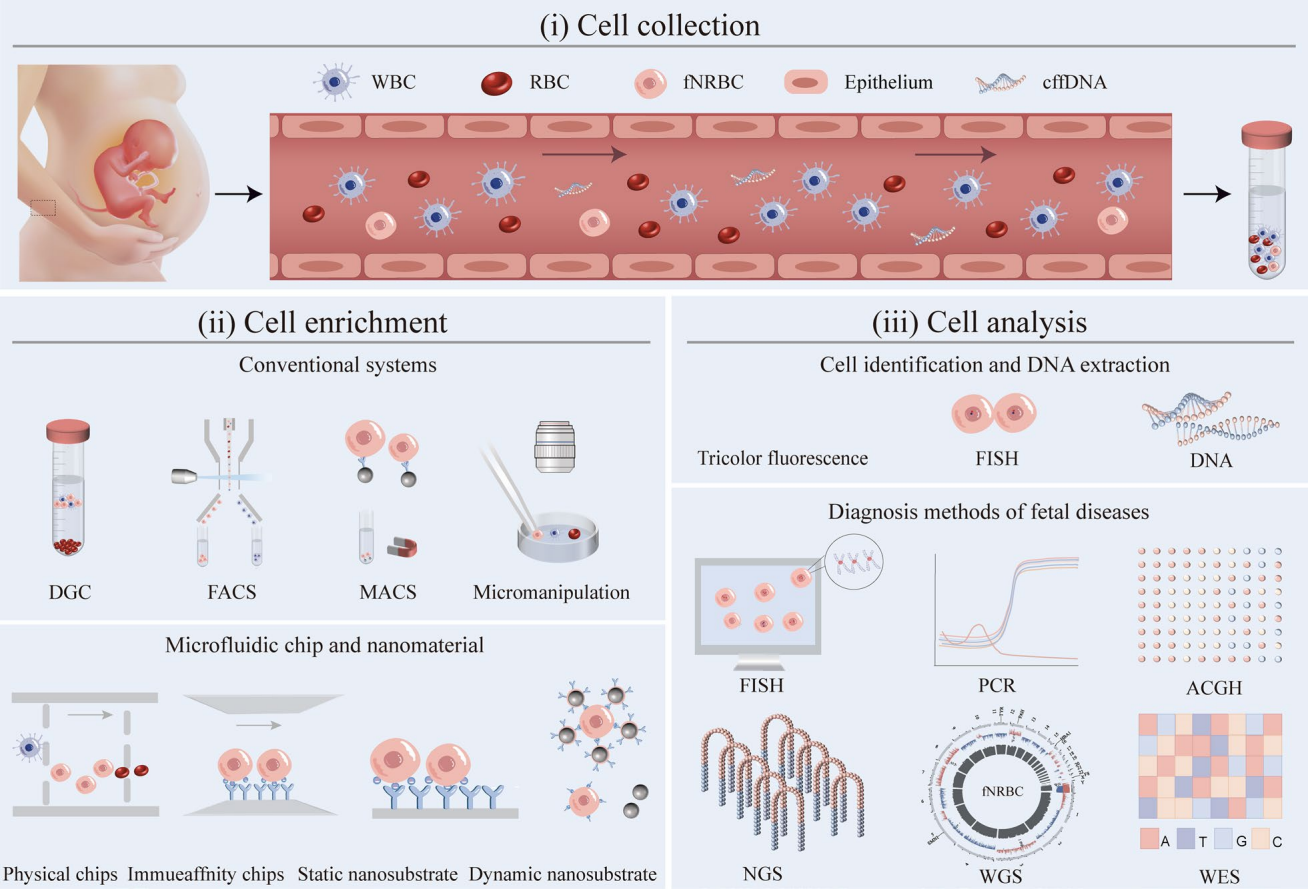
Schematic working flow of noninvasive clinical diagnosis using fNRBCs: (i) Collection of fNRBCs; (ii) Enrichment of fNRBCs; (iii) Analysis of fNRBCs

Fetal-nucleated red blood cells (fNRBCs) are considered as rare cells since there are only several to several tens of per mL of maternal peripheral blood [28]. They can be detected at 6 weeks of gestation, and their number increases with gestational weeks, reaching a peak at around 17 to 18 weeks [29, 30]. It has also been shown that the number of fNRBCs is related to the sex of the fetus, and male fetuses generally have a higher amount of fNRBCs than female fetuses [13]. Additionally, the number of fNRBCs may be associated with certain diseases. It was found that fetal hypoxia and anemia could be caused by factors like fetal prematurity, chronic hypoxia, ABO hemolysis, maternal diabetes, maternal smoking, and congenital TORCH infections (toxoplasma, other pathogens, rubella, cytomegalovirus, herpes simplex virus). These factors could contribute to the elevation of erythropoietin, thus leading to a pathological increase in the number of fNRBCs [31]. Even though fNRBCs have numerous distinctive advantages, NIPD targeting fNRBCs is limited by the separation of fNRBCs due to their extremely low number presenting in maternal peripheral blood [32]. Conventional cell separation and enrichment methods such as density gradient centrifugation (DGC) [33], fluorescence-activated cell sorting (FACS) [34], and magnetic-activated cell sorting (MACS) [35] have been used for fNRBC isolation. However, these methods are mainly suffering from the purity and sensitivity issues. With the advantages such as high throughput, high sensitivity, integration, and miniaturization, micro-/nanotechnologies have attracted great attention for isolating fNRBCs [36]. Micro-/nanomaterials can improve the cell capture rate compared with conventional methods because the size of micro-/nanomaterials matches the cell better and provide more surface area for binding ligands. Meanwhile, nanostructured substrates can retain the integrity and activity of the cells, enabling the subsequent identification and analysis of fNRBCs for NIPD (Fig. 1) [37].

NIPD targeting fNRBCs may hold great potential as treatment guidelines for clinics. Currently, FISH, short tandem repeat (STR), PCR, array comparative genomic hybridization (ACGH), next-generation sequencing, and whole-genome sequencing (WGS) are used to diagnose fetal diseases. FISH and STR can identify the origin of cells and detect aneuploidy diseases caused by multicopy number variants of fetal chromosomes [38, 39]. PCR techniques can detect single gene disorders caused by single nucleotide variants [40]. ACGH techniques can further detect chromosomal microduplications, microdeletions, micro-rearrangements, and abnormalities caused by sub-microstructures [39]. In addition, single nucleotide insertions, deletions, and other variants need to be analyzed in combination with NGS, WGS, and WES [41–43]. For clinical use, the physician needs to choose the specific analysis technique in the context of the pregnant woman. The techniques targeting fNRBCs may provide comprehensive genetic information about the fetus and great support for the application of NIPD.

## Conventional separation/sorting methods for FNRBCs

It is challenging to isolate fNRBCs from maternal peripheral blood due to the limited and variable cell numbers. The current common isolation methods are based on the size, deformability, and density of fNRBCs as well as the surface antigens. These strategies include DGC [33], FACS [34], MACS [35], affinity lectin separation method [44], and single-cell microscopy separation method [45]. The advantages and disadvantages of these methods have been illustrated in Table 2. Notably, several methods are often used in conjunction with capturing cells with higher purity and better cell viability.

**Table 2:** Merits and Demerits of Different Methods for Isolating FNRBCs

Technology	Key Features	Merits	Demerits	References
Conventional	Cell size;	Simple;	Low capture rate;	[32]
	Cell density;	Low-cost to operate	Low purity;	
	Surface antigen		Low vitality;	
DGC	Cell density	Simple;	Low purity;	[33]
		Low-cost to operate	Pretreatment method	
FACS	Surface antigen;	Fast;	Expensive;	[34]
	Fluorescent fuels;	High purity;	High requirements	
	Flow cytometry	Sort multiple cells Simultaneously		
MACS	Surface antigen;	Convenient;	Low purity;	[56]
	Magnetic bead;	Less costly;	Not sorting multiple cells	
	Magnetic Fields	Wide application		
Affinity lectin separation	Galactose residue;	Low cost;	Low purity	[44]
	SBA	Simple;		
		High capture rate		
Microscope operation	Morphology of stained cells	Convenient;	Expensive;	[60]
		For single-cell sorting	High demanding	
Novel	Micro-/nanomaterials;	High sensitivity;	Few clinical applications	[64]
	High throughput;	High capture rate;		
	Miniaturization	High purity;		
		High vitality		
Microfiltration chips	Cell size;	Not require biomarkers;	Cell clogging;	[85]
	Cell deformability	Simple to operate;	Low purity;	
		High throughput	Low vitality	
DLD microchips	Cell size;	Not require biomarkers;	Large blood samples;	[91]
	Displacements and directions;	Simple to operate;	Cell clogging;	
		High capture rate;	Low purity	
DEP microchips	Dielectric properties;	Simple to operate;	Electrodes easily electrolyze;	[92]
	Inhomogeneous electric field;	For single-cell sorting	Generate some air bubbles;	
	Displacements and directions		Long sorting time	
Acoustic chips	Cell size;	Non-contact manipulability;	Demanding equipment;	[130]
	Acoustic contrast factors	High biocompatibility;	Complex sorting	
		Gentleness		
Droplet chips	Incompatible multiphase fluids;	Miniaturization;	High cost;	[144]
	Micro-valve control;	Confinement;	Few clinical application	
	Micro-sized droplets	Parallelism		
Immunoaffinity microchips	Surface antigen;	High capture rate;	Few clinical applications	[150–152]
	Immunoaffinity	High purity;		
		High vitality		
Static nano-substrates	Surface antigen;	Easy to operate;	Non-specific cells adhesion	[30, 39, 154, 173]
	Immunoaffinity;	High capture rate		
	Nanomaterials;			
	Nanomembranes			
Dynamic nano-substrates	Surface antigen;	Reduce WBCs adhesion;	Microbeads easily cluster	[14, 28, 38, 170]
	Immunoaffinity;	Simple and low cost;		
	Magnetic beads	High throughput		

### Density gradient centrifugation (DGC)

The DGC method is used to obtain the needed cell layers by centrifugation according to the differences in density, volume, and deformability of each cell type. The density of fNRBCs is about 1.077 to 1.130 g/mL, mature erythrocytes are approximately 1.090 to 1.110 g/mL, and leukocytes are from 1.084 to 1.088 g/mL. The diameters of fNRBCs are about 9 to 13 $\mu m$, leukocytes are approximately 7 to 20 $\mu m$, and mature erythrocytes are from 6 to 8 $\mu m$. Despite the diameters of fNRBCs overlapping with those of leukocytes [46], the unique range of density can distinguish between cell types. DGC is divided into single-DGC, double-DGC, and triple-DGC. Samura et al. [47] used single-DGC to isolate fNRBCs and compared different densities of Histopaque buffer and found that 1.19 g/mL buffer was better than 1.090 g/mL. However, they collected a few fNRBCs mixed with many leukocytes and platelets, leading to a low enrichment rate of target cells and low cell purity. To improve cell purity, Jeon et al. [33] enriched fNRBCs from maternal peripheral blood using 1.077 g/mL and 1.119 g/mL Percoll for detection of fetal sex and aneuploidy. In addition, Ganshirt-Ahler et al. [48] successfully achieved enrichment of fNRBCs using a triple-DGC method for the detection of T21 and T18. This method was shown to have a higher cell enrichment rate than the single-DGC method. The volume of cells changes when they are captured, which may influence downstream purity. This led one researcher [49] to maintain the volume of the cell at high osmotic pressure, later combining it with DGC to sort the target cells. They found an ideal osmotic pressure by comparing the capture efficiency of target cells with different osmolarities and successfully isolated fNRBCs. This method improved the purity of cell separation. DGC is simple, low-cost, and easy to operate. To a certain extent, it can achieve the effect of cell enrichment. However, this method cannot separate several types of mononuclear cells well, which makes the isolated fNRBCs less pure and smaller in number and would harm the downstream analysis and detection. Thus, this method is generally only used for pretreatment experiments of target cells.

### fluorescence-activated cell sorting (FACS)

FACS is a cell sorting method based on the binding of the surface-specific antigens of target cells with fluorescein-labeled probes by flow cytometry. Extensive studies have demonstrated that fNRBCs have distinct cell markers, such as CD71, CD36, and GPA [26, 50–52]. According to these biomarkers, scholars have used flow cytometry to count, isolate, and enrich target cells, which has greatly improved the efficiency of the enrichment process [34, 50, 51]. The number of target cells using this method is mainly related to the chosen type of antibody. Bianchi et al. [26] successfully isolated fNRBCs from the peripheral blood of 49 pregnant women by using flow cytometry based on three antibodies including anti-CD71, anti-CD36, and anti-GPA. The results showed that cells isolated using GPA antibodies were 100% accurate for sex prediction and their enrichment of cells was more efficient than other antibodies. In addition, Ito et al. [34] successfully isolated target cells by using a positive selection FACS system with two erythrocyte markers CD71 and Mouse anti-Human CD235a. The captured cells were then lysed to obtain DNA and subjected to labeling Y-chromosomes by polymerase chain reaction (PCR) and whole genome amplification (WGA) techniques, allowing researchers to find the origin of the cells. Yurtcu et al. [53] obtained specimens from the cervix of 100 pregnant women and isolated target cells by FACS and MACS in parallel using antibodies corresponding to human leucocyte antigen-G (G233) and placental alkaline phosphatase (PLAP). When using the FACS system, the data showed that the percentage of positive cells for HLA-G233 and PLAP was 4.55% and 84.59%, respectively, and 14.75% in combination with both. Considering that fNRBCs have high expression of hemoglobin F (HbF), whereas adults have high expression of hemoglobin A (HbA). Bohmer et al. [52] successfully isolated fNRBCs based on this differential expression of fetal and adult hemoglobin. They first cultured the cells from maternal peripheral blood and then isolated fNRBCs using two-color fluorescent labels with this expression differential, achieving 50% sorting purity. All these methods above use positive selection to capture target cells, so there will be a large number of non-specific cells, which hinders the downstream analysis. Therefore, in future studies, a combination of positive and negative approaches is required to improve the purity of specific cells. Although the cell capturing efficiency of this method is more efficient than that of DGC, it is relatively demanding on complex operations and expensive instruments; besides, cell capturing efficiency is antibody-dependent, so it is rarely used in clinics.

### Magnetic-activated cell sorting (MACS)

MACS is a method of magnetically sorting target cells based on the combination of cell surface-specific antigens and antibodies coated on magnetic beads. The MACS-based method is convenient, inexpensive, and widely used in rare cell isolation studies. Zheng et al [54]. collected 52 fetal villi and maternal peripheral blood samples and used DGC and MACS to sort GPA-positive cells out and cultured them, and then identified the captured cells by FISH and PCR techniques. In addition, Ganshirt-Ahlert et al. [48] used a triple-DGC (1.077, 1.110, 1.119 g/mL) Histopaque system to centrifuge peripheral blood samples from pregnant women, later sorting fNRBCs by the MACS system. This method improved cell purity and capture efficiency. Based on this method, Fukushima et al. [55] optimized the method by combining a double-DGC (1.077, 1.119 g/mL) Histopaque system, and MACS to achieve the fNRBC separation. They collected the centrifuged mononuclear cells using a syringe, modified the cells with anti-CD45, incubated the cells with goat anti-mouse IgG magnetic beads, sorted the cells using the MACS system, and identified the origin of cells with the help of the PCR technique. The results demonstrated the feasibility of collecting fNRBCs from the peripheral blood of pregnant women and using them for NIPD. To compare the performance of MACS and FACS, Nemescu et al. [56] used both methods to capture fNRBCs from 27 males and identified the cells with FISH techniques. The results showed that the paramagnetic hemoglobin technique isolated significantly more fNRBCs than the anti-CD71 technique did. This suggested that the MACS technique was more efficient in enriching cells than the FACS technique. While MACS is easy to operate, fast in sorting, and broad in applications, the purity of fNRBCs is generally low due to its dependence on the antibodies used. In addition, the added magnetic beads are not easy to isolate, which affects the detection and analysis of cells.

### Affinity method

It has been found that both fNRBCs and RBCs express galactose residues. Specific enrichment of fNRBCs can be achieved by adhering fNRBCs to galactose polymer substrates through soybean agglutinin (SBA) containing specific galactose agglutination. Kitagawa et al. [44] used this method to successfully isolate fNRBCs in 96% of the pregnant women's peripheral blood with an average of 7.8 cells per 7 mL of blood. Later, by using FISH, they detected that 7 out of 8 male cells contained chromosomal sex-determining genes and confirmed that more than 50% of the isolated fNRBCs were of fetal origin. Compared with FACS or MACS, this method is low-cost, captures more cells, and does not require the addition of magnetic beads and antibodies, so it has the advantage of easy implementation. To identify the differences between this isolation technique and MACS, Babochkina et al [57]. Sorted fNRBCs in parallel using both SBA and MACS methods. The results showed that the number of cells collected using the SBA method was eight times higher than that of MACS. Compared with MACS, it had better recovery rates and morphology of the enriched cells. Although this method can recover many cells, the collected cells are mixed with a large number of maternal cells, thus reducing the purity of the cells. To solve this problem, Kanda et al. [58] used a combination of an automated identification and recovery system for fNRBCs and a leukocyte negative selection method to improve the purity of cells. They first pretreated peripheral blood samples from 39 pregnant women using the DGC method and recovered leukocytes using anti-CD45. The target cells were then attached to the slide by combining galactose-specific lectin and galactose-binding vinyl polymers, and then the fNRBCs were recovered using an automatic identification system after staining. They used a laser capture microscopy cutting technique to isolate single nucleated RBCs from 8 male fetuses, extracted DNA, and identified the origin of the cells using PCR and FISH methods. The results showed that fNRBCs were successfully identified using automatic identification technology, with an average of 18 to 6000 fNRBCs in 10 mL of blood. They then isolated 71 target cells from maternal blood samples of 8 male fetuses and detected 7 of the 8 samples containing the Y chromosome by FISH. The method increased both the number of cells captured and the purity of the target cells. These combined methods of leukocyte negative selection, automatic target cell identification system, and lectin, which do not require the addition of magnetic beads, are low cost, efficient, and provide a novel approach for NIPD.

### Microscope operation method

The microscope operation method utilizes the specific morphology of fNRBCs after staining and processing under the microscope to recover fNRBCs using a micro-manipulator. This technique can obtain individual cells directly under the microscope, which avoids contamination of other monocytes. It can facilitate downstream cell analysis, noninvasive prenatal testing, and the prognosis of the postnatal disease. As early as 1995, Takabayashi et al. [59] stained cells using the Pappenheim method. The stained fNRBCs were small in size, dark in color, with no particles in the cytoplasm, and a nucleus biased to one side [60]. The cells were collected based on morphological analysis of stained cells using a microscope manipulator. Next, Sekizawa et al. [61] first isolated monocytic layers using a double-DGC, followed by Giemsa staining. According to the morphology of the cells, cells with a low nucleoplasmic ratio and no particles in the cytoplasm were recovered using a micro-manipulator. Compared with other Conventional methods, a larger number of target cells were obtained, avoiding more waste, and allowing more target cells to be obtained. In addition to the use of chemical staining, other methods have isolated different subtypes of fNRBCs using fluorescent labeling of specific proteins expressed by fNRBCs. Nagy et al. [62] used MACS and micro-manipulation techniques to isolate fNRBCs expressing ε-hemoglobin chains and used them to identify the sex of the fetus. Later in 2016, Giambona et al. [60] isolated fNRBCs from 42 samples of body cavity fluid using 40 × optical phase-contrast microscopy. Using a micropipette, they isolated a single target cell based on the diameter of the cells (12–16 m) and changes in cell morphology after staining. Although individual cells can be isolated, the technique is limited by the highly demanding operation. Based on these problems, an automated microscope image analysis capture system has been proposed, which reduced the technical requirements. For example, Oosterwijk et al. [63] used an automated microscopic image analysis capture system to isolate fNRBCs from the peripheral blood of 42 pregnant women and compared the differences between manual detection and automated microscopic detection. The system included enrichment, chemical staining, and FISH methods to identify chromosomal SRY genes. The results showed that 52% of the slides were positive for HbF using the automated microscopy technique, compared to 43% when using the manual detection technique. It illustrated the advantages of automated microscopy screening over manual screening. In addition, the automated screening system analyzed the DNA of 11 male cells using the FISH method and diagnosed 7 cases containing the SRY gene. It showed the feasibility of this method. This technique saves on labor costs and reduces the workload. Compared with other staining-based microscopic manipulation separation techniques, the automated microscopic image analysis and capture system uses specific antibodies to capture the target cells with much higher specificity and capture rate. In addition, cells can be detected, and the resulting image information can also be stored for later verification, which is better than flow cytometry sorting.

These conventional separation methods are simple, easy to operate, low cost, and can enrich cells quickly to a certain extent. To improve the capture efficiency and purity of cells, the above methods can be used in combination. However, these methods cannot effectively distinguish fNRBCs from other cells with differences in size, density, and antigen expression. They can also lead to the contamination of target cells due to factors such as magnetic beads not being easily separated, which makes the purity and activity of the cells generally lower. In addition, considering the rarity of fNRBCs and the presence of many background cells, the cells enriched using these methods can cause significant interference for downstream FISH analysis. Therefore, it is urgent to identify a method that can achieve high efficiency, high purity, and high activity capturing.

## Separation methods based on micro/nanotechnologies 

In recent years, with the rapid development of micro-/nanotechnology, the cell separation methods based on micro-/nanotechnologies have attracted great attention. We mainly discussed the microfluidics and nanomaterial/devices (Tables 2 and 3) for improving the current conventional methods.

**Table 3:** Novel methods for microchips and nano-substrates

Methods	Sorting technology	Release	Efficiency /Count	Advantages	Disadvantages	Analysis method	Diagnostic outcomes	References
Physical chips	A microfiltration chip containing	Filtration	1.2/mL	Simple and low cost	Low purity	—	—	[83]
	micropores of different widths				Low vitality			
	A cross-flow filtration chip	Filtration	Capture: ~ 74.0%	Simple and low cost	Low purity	—	—	[85]
					Low vitality			
	A DLD chip with magnetic	Filtration;	37.44/mL	High throughput;	Chip clogging	PCR	Gender	[91]
	columns	Magnetic Fields		High purity			Identification	
	A two-step cascade DLD chip	Filtration	1–396/mL	High purity	Reduce cell yield	PCR	Gender	[82]
	with RBC-positive enrichment	Magnetic Fields	Purity: ~ 87.8%				Identification	
	A DEP chip containing	Electric Fields	—	Automation;	Long sorting times;	—	—	[92]
	electrodes, sensors,			Single-cell sorting	Low efficiency;			
	and microchambers				Electrode contamination			
	A BAW-based acoustic chip	Sound waves	Capture: ~ 40.64%	High biocompatibility;	High requirements;	PCR	—	[130]
				Mildness	Complex operation			
	A droplet microfluidic chip with	Valves	Vitality: ~ 99%	High viability;	Not easily release cells;	FISH	—	[144]
	calcium alginate hydrogel			On-chip analysis;	Target cell loss			
	particles			Single-cell analysis				
Immunoaffinity chips	A hydroxyapatite/chitosan chip	In-situ	1420–3221/mL	Biocompatible;	Not release cells;	FISH	T13/T21	[150]
	modified with anti-CD147			High throughput	Non-specific cells adhesion			
	A triangular micropillar chip	In-situ	Capture: > 90%	High capture rate	Not release cells	FISH/qPCR	—	[151]
	with CD71 antibody-labeled		5–35/2 mL					
	A fishbone microfluidic chip	MMP-9	Capture: > 80%	Easily release cells;	Complex production;	FISH	T21/T13	[152]
	with gelatin and anti-CD147		Release: ~ 89%	High throughput;	Target cell loss		XXY/XXX	
			Purity: ~ 85%	High capture rate;				
			Vitality: > 90%	High purity				
			3–24/mL					
	A two-stage integrated-decision	In-situ	Release: > 85%	Single-cell analysis;	Not release cells;	FISH	—	[153]
	grading platform			High throughput;				
				High recovery rate				
Static nano-substrates	A polypyrrole nanoparticles	Voltage	Release: ~ 94.6%	Biocompatible;	Affect cell vitality;	FISH/WES	T21/T13	[30]
	the film with biotin and anti-CD147		22–58/mL	Easily release cells	Non-specific cells adhesion		/T18/XXY	
							/Microdeletion	
	Chitosan nano-substrate with	DTT	Capture: ~ 90%	Damage-free release;	Non-specific cells adhesion	FISH	—	[154]
	NHS-(S–S)-Biotin and		Release: ~ 90%	High recovery				
	anti-CD147		Vitality: > 91.2%					
			7–26/mL					
	A "Cell Reveal™" platform	In-situ	14–22/4 mL	Automated system;	High demanding;	FISH/STR	T21/T13/T18	[39]
				Easy to operate;	Non-specific cells adhesion	NGS/aCGH		
				On-chip analysis				
	A coral-like in silico platform	In-situ	Capture: 88.1%	Automated system;	High demanding	FISH/STR	T21/T18	[173]
			Release: 90%	High capture rate;		NGS/aCGH	/microdeletion	
			2–71/2 mL	Easy and low cost;				
				On-chip analysis				
Dynamic Nano-substrates	SiO_2_ microbeads modified with	Magnets	42–93/mL	Easy and low cost;	Not easily release cells;	FISH/PCR	ABO blood grouping	[28]
	SA and anti-CD147			High throughput	Low recovery rate			
	Size-scaled silica spheres with	MMP-9	Capture: 81%	Enlarged the size;	Microbeads easily cluster	FISH/PCR	—	[14]
	gelatin coating and anti-CD147		Release: 80%	A harmless release;				
			Purity: 83%	High recovery				
			7–65/mL					
	SiO_2_@MnO_2_ microbeads with	Oxalic acid	Capture: ~ 80%	Easy and low cost;	Microbeads easily cluster;	FISH/STR	—	[38]
	anti-CD147		6–32/mL	Biocompatible	Not easily release cells			
	Magnetic nanoparticle coated	Magnets	Capture: 90%	Against WBC adhesion;	Not easily release cells	FISH	T21/T18/XXX/	[170]
	with a mixed membrane of		Release: > 80%	High capture;			XXY/XYY	
	leukocytes and erythrocytes		Vitality: > 80%	High purity				
			Purity:87%					
			10–30/mL					

### Microfluidic separation methods

Microfluidics, also called lab-on-a-chip, has recently been used extensively in the biomedical field. The chips can concentrate cell separation, biochemical reactions, and assay analysis in micro-/nanoscale pores, which facilitates cell separation as well as downstream analysis [14, 64–77]. According to the research needs, the internal structure of chips can be designed to achieve precise control of the fluid [65, 78, 79]. The chip can be fabricated by techniques such as photolithography, 3D printing, and molding methods [80]. With the development of this technology, its internal structure tends to be increasingly refined, and chips synthesized using polydimethylsiloxane (PDMS) are now more widely used [80–82]. An increasing number of researchers are using microfluidics to isolate fNRBCs. Depending on the characteristics of the target cells, the main methods include physical and biochemical immunoaffinity methods.

#### Microfluidic physical methods

**Fig. 2:**
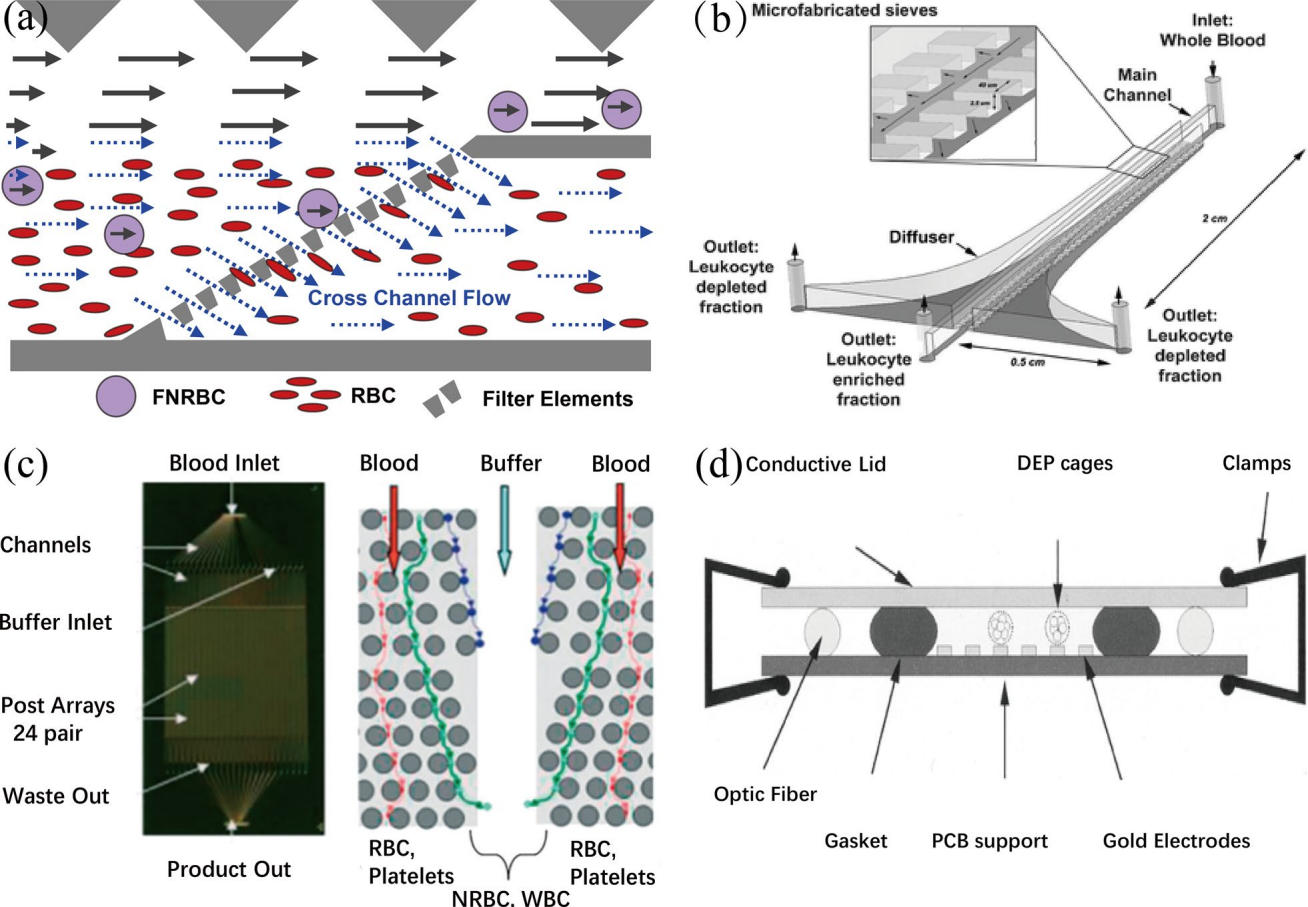
Microfluidic physical methods for sorting/separation of fNRBCs. **a** Cross-flow microfiltration chip for fNRBC collection. Reprinted with permission from ref [85]. Copyright 2010 Elsevier. **b** Diffusion filters for continuous flow classification and separation of fNRBCs. Reprinted with permission from ref [86]. Copyright 2006 The Royal Society of Chemistry. **c** Determining lateral displacement microfluidic chips: the magnetic bead microarrays for a two-step process for the separation of fNRBCs. Reprinted with permission from ref [91]. Copyright 2008 John Wiley & Sons*.* **d** Dielectrophoretic chips: the automated capture method by generating DEP cages to capture fNRBCs. Reprinted with permission from ref [92]. Copyright 2003, IEEE

The physical methods have been developed for cell separation based on the size, deformability, and density of cells. Here, we discuss the fNRBC separation methods (Fig. 2) including microfiltration method, deterministic lateral displacement (DLD) chip, dielectrophoresis (DEP) chip, acoustofluidics, droplet microfluidics, and others.

##### Microfiltration chips

The microfiltration method [83] is used to isolate target cells based on the difference in the size and deformability of various cells. Mature erythrocytes are smaller in size than leukocytes and fNRBCs, and there exists a partial overlap between them. Compared with leukocytes, fNRBCs have better deformability, so chips with various pore sizes can be prepared to achieve target cell separation. Common microfilters include column, cross-flow, weir, and membrane filters [84]. Mohamed et al. [83] designed a chip containing a series of micro-columnar structures of varying widths based on the differences between leukocytes and fNRBCs, which had narrow microcolumns of 15, 10, 5, and 2.5 $\mu m$ in sequence along the direction of the sample cell flow. The results showed that leukocytes with a larger size and poorer deformability failed to pass through the smallest size microcolumns and would linger nearby. In contrast, fNRBCs with smaller sizes and better deformability could pass through the 2.5 $\mu m$ microcolumns, thus enabling the separation of fNRBCs. This method does not require modifying antibodies on the chip to isolate cells from blood and is simple to operate and prepare. Since this chip has multiple channels, experimental time is increased, more blood samples are required, and the efficiency of capturing cells is lower. In addition, there will be a blockage of the channels by cells during the experiment, which results in the failure of fNRBCs to flow further toward the small-sized channels and a decrease in the number of captured target cells. Lee et al. [85] compared column, cross-flow, weir, and membrane microfilters and showed that the cross-flow chip was more suitable for the separation of whole blood cells. Thus, they designed a two-in, two-out cross-flow microfiltration chip (Fig. 2a). The cross-flow filtration device was prepared with a row of microcolumn in the direction of fluid movement, forming (Table 4) a relatively narrow slit. The smaller-sized mature RBCs entered the slits, while the larger-sized fNRBCs followed the direction of the flow into the collection channel, thus collecting the target cells. The results showed that 46.5% of the mature erythrocytes were filtered and 74.0% of the target cells were enriched. Even though the chip can filter out mature erythrocytes and reduce cell clogging, the collected cells still contain a large number of leukocytes. To reduce the interference of leukocytes, some researchers used the cross-flow microfiltration method to separate leukocytes and thus removed them from whole blood, which provides a new idea for high purity enrichment of fNRBCs (Fig. 2b) [86, 87]. The microfiltration-based method does not require special labeling, and the equipment is easy to operate. An overlap in size exists between all the cells, leading to the purity of the cells obtained by separation being low, which therefore also leads to chip clogging. In addition, the cells were subjected to fluid shear during experimentation, which can lead to a decrease in cell activity.

**Table 4:** Clinical application of FNRBCs

Technology	Variation Type	Merits	Demerits	Fetal Diseases	References
FISH	Chromosome multicopy	Simple operation;	Fetal origin identification is	T13/T18/T21	[39]
	number variation	Fetal origin identification	applicable to male fetuses; Male fetuses		
			Results are influenced by cell		
			purity		
STR	Chromosome multicopy	Fetal origin identification;	Fetal origin identification is more	T18/T21	[187, 189, 190]
	number variation	Not limited to male fetuses;	complex		
		High sensitivity			
PCR	SNVs	High sensitivity;	High demanding;	Sickle cell anemia;	[40]
		High specificity;	Prone to false positives	ABO blood group;	[28]
		Simple and fast		T18/T21	[194]
ACGH	CNVs of genes	High resolution;	Detection of some unknown	T13/T18/T21;	[39, 201]
	(Chromosomal multi-copy	Without culture;	significance of CNVs	Rearrangement variants	[198]
	Number, variation,				
	microdeletions,				
	microduplications,SVs)				
	Chromosomally				
	unbalanced variants				
NGS	CNVs of whole genes	High-throughput;	High cost;	T18/T21/MMS;	[42, 212]
	Chromosomally	Comprehensive Analysis	Detection of some unknown	Congenital	[41]
	balanced variants		significance of CNVs	Deafness	
WGS	CNVs of whole genes	High-throughput;	High cost;	Single gene	[222]
	(SNVs/In Dels/CNVs/SVs)	More comprehensive	Detection of some unknown	disease	
		genetic information	significance of CNVs;		
			Low coverage will miss variants		
WES	CNVs of whole exon genes	High-throughput;	High cost;	13/18/21	[30]
	(SNVs/In Dels/CNVs/SVs)	Small sequencing range	Detection of SNVs is not as	18q21s	
			reliable as WGS	microdeletion	

##### Determined lateral displacement (DLD) chips

The DLD method [88] can separate target cells based on differences in displacement and orientation of different cell types within the chip due to the variability of their sizes. When cells flow within DLD chips, they will encounter the array and show an asymmetric bifurcation of flow around it, and cells of similar size will have a similar path [89]. Smaller erythrocytes and platelets will always flow with the stream eventually to the corresponding outlet, while larger fNRBCs and leukocytes encountering the micropillar will leave the stream in a small angular direction. In 2004, Huang et al. [90] prepared DLD microfluidic chips based on this property. They designed nanoscale asymmetric microarrays for the continuous separation of cells and set up a high flow rate fluid to reduce cell diffusivity, thus obtaining a more defined cell path for collecting cells. This method improved the yield of target cells, but the purity of the obtained cells was low. In 2008, Huang et al. [91] further optimized the method by preparing asymmetrical magnetic microcolumns based on the variability of displacement and orientation of cells of varied sizes in the fluid and achieved the separation of fNRBCs with a magnetic field (Fig. 2c). They collected peripheral blood samples from 58 pregnant women and separated the cells using a two-step method. They first used microarrays to obtain cells containing the nucleus, then passed these cells through the microarrays containing magnetic columns to separate fNRBCs with hemoglobin. The hemoglobin of fNRBCs was converted to methemoglobin in magnetic columns, then had paramagnetic properties, while leukocytes were not magnetically labeled. Therefore, fNRBCs could be well separated. The results showed that each milliliter of pregnant women's peripheral blood samples contained an average of 37.44 target cells. Compared with the results of previous studies, the number of cells enriched by this method was increased by 10 to 20 times, and the purity was also higher than that of the passive separation method alone. However, such chips can cause chip clogging when handling clinical samples, and the bubbles generated by the cell suspension during the flow process may also affect the separation of target cells. Overall, this method is promising for clinical applications because of the higher number and purity of target cells obtained compared to other methods.

##### Dielectrophoretic (DEP) Chips

Dielectrophoretic is a phenomenon in which various particles undergo different orientations and displacements in an inhomogeneous electric field. Different cells have different dielectric properties and undergo different displacements and orientations. Based on this dielectrophoretic variability of cells, researchers proposed a DEP chip to separate target cells. Xu et al. [88] designed a DEP chip containing crossed microelectrodes and microchannels to capture fNRBCs. The crossed electrode array could generate an inhomogeneous electric field, and various cells were distinguished by different external forces in the electric field because of the differences in dielectric properties. The results demonstrated that the chip could be used for the capture of fNRBCs. The method does not require biomarkers to obtain cells with high activity. However, considering that the metal electrodes are directly exposed to air and can be easily electrolyzed, and some air bubbles will be generated during the experiment, these factors would have an impact on the sorting of target cells. If the blood is not treated in advance, it may also contaminate the electrode. In addition, there is a crossover in the dielectrophoresis of various cells, so the purity of the separated cells is not high, and secondary treatment is usually required. These pose great difficulties in obtaining target cells with high purity. To improve the purity of the target cells, reduce contamination, and achieve single-cell sorting, Medoro et al. [92] designed a DEP chip containing electrodes, sensors, and microchambers. The device could be automated with software tools for sensing and driving operations. The presence of positive and negative sinusoidal voltages allowed the fluid passing through the microarray to form a small, closed DEP cage above it (Fig. 2d). Single or multiple cells were captured in the cages with the voltage being changed to control the position of the single cells. Thus, single cells were captured into separate DEP cages. This method allows for the sorting of single cells and quantification of the collected cells, thus greatly improving cell purity and capture efficiency. Borgatti et al. [93] further optimized this method. They designed a two-dimensional microarray chip that produced columnar and spherical DEP cages to capture the K562 cell line, respectively. The cells express CD71 and GPA and can be used as a simulation experiment for capturing fNRBCs [94]. The results showed that the platform could move individual cells into a single DEP cage. In addition, it was also verified that a single microsphere and a single target cell could achieve efficient binding in the same DEP cage under software control. The system can separate target cells in an all-electronic system without the need to control the flow of fluid, resulting in significant cost savings, and immense potential for subsequent analysis of single cells as well as for drug delivery by target cells. Current DEP chips still have difficulty for clinical applications due to some technique barriers such as contamination of electrodes, long sorting times, low efficiency of sorting, and low cell purity. Therefore, an automated DEP chip may be integrated with the isolation, release, and lysis of single cells, as well as downstream analysis for fNRBCs.

##### Acoustofluidic methods

Acoustofluidics [95–109] through the fusion of acoustic waves and microfluidics has been used for cell separation based on the size, shape, and acoustic contract of the cells, due to their advantages including contactless manipulation, label-free operation, and high biocompatibility [110–125]. Recently, the Huang group made pioneer efforts on developing surface acoustic wave (SAW)-based microfluidics for the separation of rare cells [126–129]. Moreover, Wang et al. developed a bulk wave (BAW)-based microfluidic chip for the separation of fNRBCs [130]. The chip included a BAW focus module and a microfluidic fluorescence-activated cell sorter (μFACS) sorting module. They first focused the cells in three dimensions using acoustic focusing. Afterward, when the cells reached the sorting zone, the target cells would be deflected toward the target exit by using pulsed acoustic waves, thus achieving sorting and enrichment of the target cells. They stained the captured cells with propidium iodide and found that the cell viability decreased by only 0.5%, indicating the high cell viability of the method. In clinical blood sample capture experiments, they finally succeeded in obtaining fNRBCs from the maternal peripheral blood and found that 40.64% of fetal cells were captured by genetic analysis. This provides a new idea for the NIPD for fNRBCs. Compared with other label-free microfluidic sorting methods, this method has high biocompatibility, high controllability, and low cell damage, while efforts are still required for improving their specificity.

##### Droplet microfluidics

Droplet microfluidics [131–136] can generate and manipulate discrete picolitre to nanoliter droplets through immiscible multiphase flows inside microfluidic channels, and have attracted extensive attention in the field of cell separation and analysis due to its advantages of miniaturization, confinement, and parallelism [100, 137–142]. One can design microchannels with different functions according to the requirements while manipulating droplets with the help of external forces to achieve better separation of target cells from other cells [143]. Sun et al. designed a droplet microfluidic chip with calcium alginate hydrogel particles for capturing the target cells and undamaged release [144]. They parallelized two focused microstructures to a chip, after forming different droplets that encapsulate cells, and the two fused downstream to form cell-encapsulated calcium alginate hydrogel particles. The chip included a pneumatic micro-valve control structure that allowed for the capture and release of the hydrogel by air pressure. In addition, they used FDA/PI staining to detect the activity of cells immobilized in the microchambers in real-time, which provided a good idea for proteomics and genomics analysis downstream of single cells. Individual dispersed droplets can be formed in the nanoscale channel, and each droplet is equivalent to a separate cell reaction laboratory, thus reducing contamination of WBCs. In addition, the chip contains a large number of microchambers, which ensures the parallelism of reactions and allows different operations to be performed in different microchambers, thus enabling high throughput analysis [136]. Thus, based on these advantages, droplet microfluidics has shown great potential in cell and particle sorting [78, 145], cell culture [65], downstream analysis of cells [79, 146], and drug delivery [147].

##### Other microfluidic physical methods

Engineering efforts have been made to develop magnetic, dynamic flow, and other integrated devices for cell separation, highlighting their applications in the fNRBC separation. To reduce the interference of WBCs. Byeon et al. [82] obtained fNRBCs using a two-step method including the erythrocyte hyper-aggregation with positive sorting and the lateral magnetophoretic micro-separator with negative sorting. They collected the peripheral blood samples from 18 pregnant women, first used the erythrocyte hyper-aggregation method to obtain monocytes containing leukocytes, and then bonded the monocyte layer suspension to leukocyte antibodies. The magnetic wires inside the chip produced a large magnetic magnitude under an applied magnetic field, and the leukocytes bonded to the magnetic beads could make a specific lateral movement along the magnetic wires, which could exit through a specific exit. In contrast, fNRBCs produced a different trajectory and exited through another exit and were thus enriched. They then used nucleic acid dyes to identify the fNRBCs and PCR to detect the SRY gene to determine the sex of the fetus and the origin of the cells. The results showed that there were 1 to 396 fNRBCs per mL of blood samples and only 0 to 6 leukocytes were identified in these target cells, with an average purity of 87.8%. This method greatly improved the purity of the target cells. Recently, Thurgood, et al. [148] developed microfluidic devices with dynamic vortices, enabling a new hydrodynamic manipulation and sorting mechanism. Along with inertial microfluidics [149], these simple, low-cost, and user-friendly devices may be used for the development of versatile and controllable microfluidic systems for fNRBCs in clinical settings.

#### Microfluidic immunoaffinity methods

**Fig. 3:**
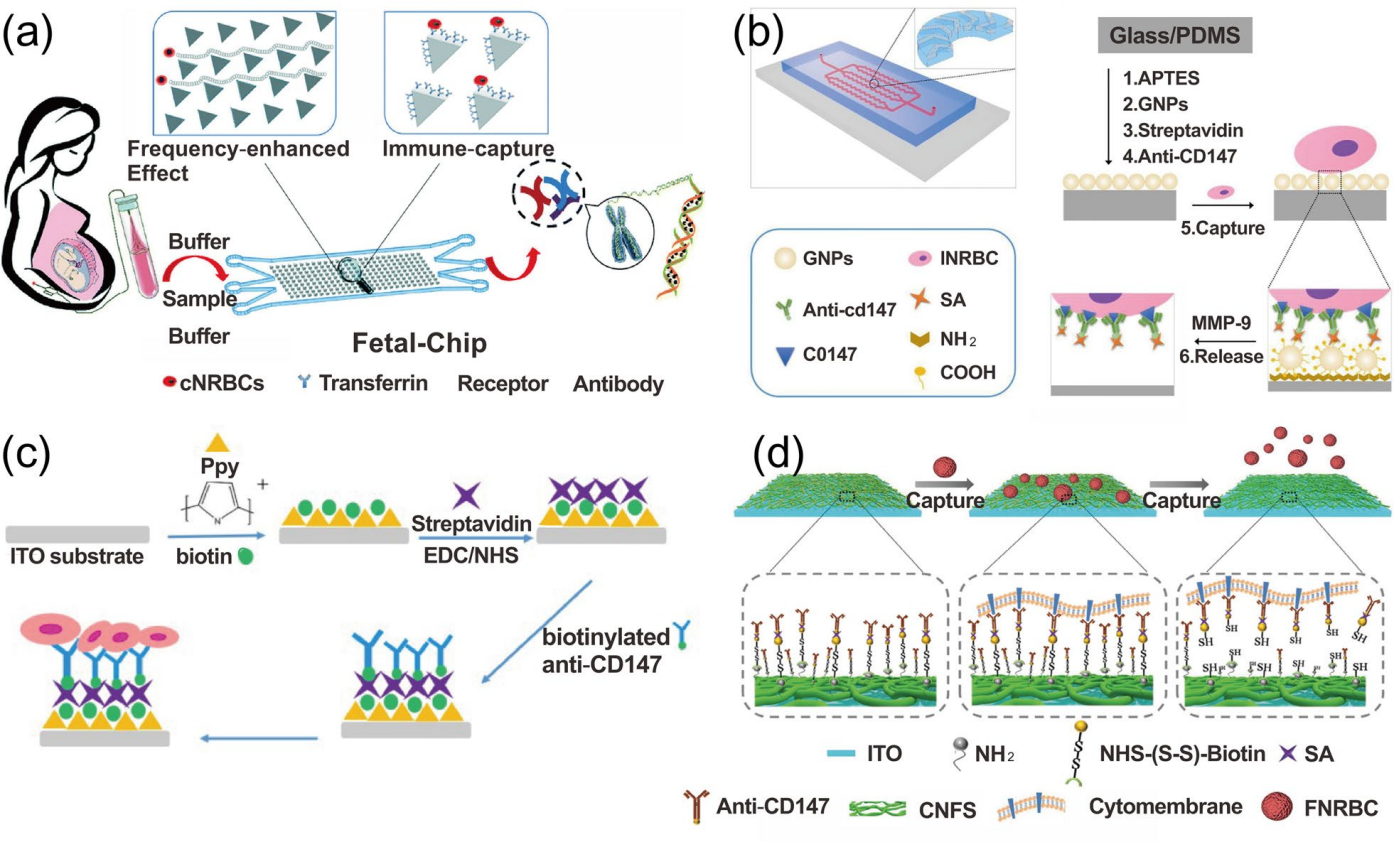
Nanostructure-embedded microchips for fNRBCs immunoadsorption methods. **a** The CD71 antibody-labeled triangular micropillar chip for sorting fNRBCs. Reprinted with permission from ref [151]. Copyright 2018 Royal Society of Chemistry. **b** The microfluidic chip with a fishbone structure modified with anti-CD147 to capture fNRBCs. Reprinted with permission from ref [152]. Copyright 2019 Elsevier. **c** Thin films of biotin-containing polypyrrole nanoparticles modified with anti-CD147 to capture fNRBCs. Reprinted with permission from ref [30]. Copyright Ivyspring International Publisher. **d** The chitosan nanostructured substrates modified with anti-CD147 to achieve sorting of fNRBCs. Reprinted with permission from ref [154]. Copyright 2020 John Wiley and Sons

The microfluidic immunoaffinity methods have shown excellent performance in capturing fNRBCs. The method is mainly based on the bioconjugation of specific antigens expressed by fNRBCs and the corresponding antibodies modified within the chip to isolate the target cells (Fig. 3). It greatly increases the capture efficiency and purity of captured cells. In 2016, He et al. designed a hydroxyapatite/chitosan nano-substrate modified with anti-CD147 to specifically capture fNRBCs [150]. They then used three-color immunofluorescence staining to verify the cells. The results revealed that the number of fNRBCs increased and then decreased with the increase of gestational weeks. At 18 weeks of gestation, the number was highest, averaging about 71 fNRBCs per mL of maternal peripheral blood. This 3D structure is highly biocompatible and provides a more specific surface area, increasing the chance of cell-chip contact and thus capturing more target cells. To further improve cell capture efficiency, researchers increase the chip-cell collision by manipulating the fluid to generate different patterns of laminar and vortex flow. Zhang et al. [151] designed a CD71 antibody-labeled triangular micropillar chip for sorting fNRBCs (Fig. 3a). The arrangement of the triangular micropillars was designed according to the DLD principle thereby generating a specific laminar flow. The laminar flow drove the large-sized cells to collide with the microcolumns, thus increasing the chance of cellular antigen–antibody binding and reducing the adhesion of background cells. The results showed that more than 90% of the target cells could be captured at a flow rate of 0.3 mL/h for cell injection. A curved channel or sudden expansion and contraction of the channel creates an unbalanced centrifugal force on the fluid, resulting in a vortex flow. Such vortexes are mainly used for sorting based on cell size differences. Wei et al. [152] designed a microfluidic chip with a curved fishbone structure and used metalloproteinases to dissolve the gelatin layer on the nanoparticles, achieving the isolation and gentle release of fNRBCs in early pregnancy. The curved fluidic channel could induce the generation of vortices to increase the interaction between cells and anti-CD147 (Fig. 3b). At a flow rate of 0.3 mL/h, 85% of the cells could be captured with a release rate of 89% and a purity of 85%. In 2020, Wang et al. [14] developed a spiral inertial microfluidic chip modified with anti-CD147 using the same release approach. Considering the effect of fluid shear on cells, they used 40 μm silica microspheres, which enlarged the size of fNRBCs and other background cells. Afterward, they relied on continuous hydrodynamic filtration generated within the chip to obtain fNRBCs and released cells by degrading gelatin with metalloproteases. They achieved high efficiency of 80%, high viability of 80%, and a purity of 83%. These vortex-based sorting methods are simple, controllable, sample low volume, and perfect for achieving cell sorting at low speeds using inertial effects. To improve the automation of the sorting system, Xu et al. [153] reported a two-stage integrated-decision grading platform for automated sorting fNRBCs, which contained optical detection and sorting collection systems. They first obtained mononuclear cells by DGC, then used the optical system to automatically detect fNRBCs expressing CD71 fluorescence positivity. Afterward, these cells were later collected by a counting filter and analyzed using immunostaining and FISH. The recovered concentration amounted to 2 × 10^6^ to 100 × 10^6^ target cells per mL of cell suspension, with a recovery rate of more than 85%. This method saves the cost of samples and antibodies, improves the purity of cells, and provides more possibilities for NIPD.

### Separation methods based on nanomaterials

The nanomaterials/devices, materials/devices with at least one dimension at the nanoscale, provide new opportunities for NIPD targeting on fNRBCs. The features of the cell surface and biological macromolecules within the cells are at the nanoscale, so nanomaterials and cells have a certain match in terms of size. It can provide a better biocompatible environment for cells or particles. In vitro, nanodevices can be connected to chemical antibodies to capture the particles we need, which can then pass these signals through sensors to a computer and perform downstream analysis. In vivo, nanomaterials can perform targeted transport of the coated drugs to the lesion, thus reducing immune rejection, and increasing the treatment efficacy of the drug. This technology has been widely used for cell sorting [154, 155], liquid biopsy [156], fluorescent labeling [157, 158], biosensors [159, 160], and target drug delivery [161, 162].

#### Nanostructured substrates for static cell separation

Nanomaterials/devices can provide more opportunities for substrates and cells to interact, which can well improve the efficiency of cell capture. As a result, an ever-growing number of researchers have fabricated nanowires [163, 164] nanotubes [165], nanodots [166], nanomembranes [145, 167, 168], and nanoparticles [38, 169, 170] for sorting rare cells. The sorting methods based on nanowires, nanotubes, nanodots, and nanofilms are static. In 2009, Wang [171] first proposed the use of a three-dimensional silicon-nanopillar array to capture rare cells. In 2016, Li et al. [164] successfully designed a cell capture and release platform with peptide-aptamer-modified nanowires to sort rare cells. In addition, Sun et al. [172] explored a multi-null titanium-nanopillar array for capturing rare circulating tumor cells. For fNRBCs, however, the most common method is based on nanomembranes. Feng et al. [30] deposited a thin film of polypyrrole nanoparticles containing biotin on conductive glass and then attached anti-CD147 to capture fNRBCs (Fig. 3c). They then released the target cells using a voltage system and detected fetal aneuploidy using FISH and whole exome sequencing (WES) techniques. Because these conductive polypyrrole nanoparticles contained biotin, they could easily release the collected cells under the effect of an electric field. In addition, these polypyrrole nanoparticles formed 3D microstructures that were compatible with the cell size, increasing the chance of contact between the cells and the chip, and thus improving the efficiency of cell capture. The results also demonstrated the feasibility of this method in diagnosing fetal chromosomal disorders. Unfortunately, the release method affected the cell viability. To solve this problem, Sun et al. [154] designed the chitosan nanostructured substrates based on electrospinning and combined with NHS-(S-S)-Biotin modified with anti-CD147 to achieve sorting as well as the undamaged release of fNRBCs (Fig. 3d). The NHS-(S-S)-Biotin molecule contained a large number of amino and carboxyl groups that could serve as a link between the substrate and fNRBCs. The Chitosan nano-basal surface was sparse, and the amino group on its surface could bind to the amino and a carboxyl group on the NHS-(S-S)-Biotin molecule, which facilitated the modification of anti-CD147 and provided more binding sites for antigen-antibody binding. They also used DTT to break the disulfide bonds to achieve damage-free release of cells and improved the chance of recovery of fNRBCs. The isolated fNRBCs were also analyzed and validated using a three-color immunofluorescence staining. Promisingly, the platform allows the isolation of fNRBCs as early as seven weeks of gestation, and the efficiency to capture target cells is greatly improved, providing a good idea for NIPD.

In addition, researchers have used a fully automated system to capture target cells. For example, Huang et al. [39] proposed a "Cell Reveal™" platform based on silicon-based nanostructures. This platform used specialized analysis software to locate and collect the target cells. The whole genome was then extracted for FISH and next-generation sequencing (NGS) analysis, which proved the origin of the cells. The feasibility of the platform to capture fNRBCs for NIPD was illustrated. To further improve the automation of the system and the efficiency of cell capture, an investigator optimized the platform by designing a coral-like *in-silico* platform to fully automate the capture of fNRBCs. The substrate of this platform was modified with fluorescently labeled anti-CD71, anti-GPA, and anti-CD45, and cells were automatically targeted for identification and counting by the automated imaging mode of the system. The counting criteria were CD71+/GPA+/CD45-/Hoechst+for fNRBCs. They then tested 14 pregnant women's peripheral blood using the platform and found a capture rate of approximately 88.1%. And 2 to 71 fNRBCs were successfully captured from all 2 mL maternal blood samples. The captured fNRBCs were then analyzed by FISH, aCGH, as well as STR, and they were confirmed to be of fetal origin. It was subsequently used to detect fetal aneuploidy, demonstrating the immense potential of the platform for application in NIPD [173]. The fully automated analysis system is simple and inexpensive to operate, and the coordinates of captured cells can be precisely mapped by taking a small amount of blood. It is certainly a step closer to a non-invasive prenatal in vitro diagnosis.

To further improve the capture efficiency of cells, microstructures can be superimposed on nanostructures to form the substrates of micro-/nanostructure, which increases the level of the substrate and achieves dual cell capture [174–176]. Therefore, Dou [176] prepared a micro/nano substrate based on the surface structure of rose petals for rare cell separation. They obtained rose petal substrates by inverse molding and attached anti-EpCAM by disulfide bonds to capture cells with high affinity. The micro-papillae of rose petals had a micrometer size from 20 to 30 $\mathrm{\mu m}$, and the surface folds had a nanometer size from 500 to 600 $\mathrm{nm}$. These structures increased the hierarchy of the substrate and facilitated the pseudopods of the cells in grasping the substrate more tightly, which greatly increased the efficiency of cell capture. The results showed a capture efficiency of 85%, followed by a non-invasive release of cells using glutathione, with an activity of up to 98% after 24 h of incubation. The natural biomaterial possesses a micro-/nanostructure that is well-matched to the cells in terms of size. The microstructure increases the contact area between the substrate and the cells, and the nanostructure provides more sites for binding to the target cells for increasing the efficiency of cell capture. Therefore, this rose petal-based sorting platform provides a promising idea for the capture of fNRBCs. Most of the above are methods of statically capturing cells, and although they can capture a larger number of cells, there will be a great number of non-specific cells adhering to the nano-substrate. In addition, the number of fNRBCs in the peripheral blood of pregnant women is very sparse, while the number of adherent background cells is large. Even though more cells are statically captured, the overall purity of fNRBCs obtained by sorting is still low, so these methods of statically capturing cells are highly limited in NIPD [155]. Therefore, we need to look for methods to capture cells with high purity, high activity, and high efficiency, which will be significant for NIPD.

#### Nanomaterial-based dynamic sorting methods

**Fig. 4:**
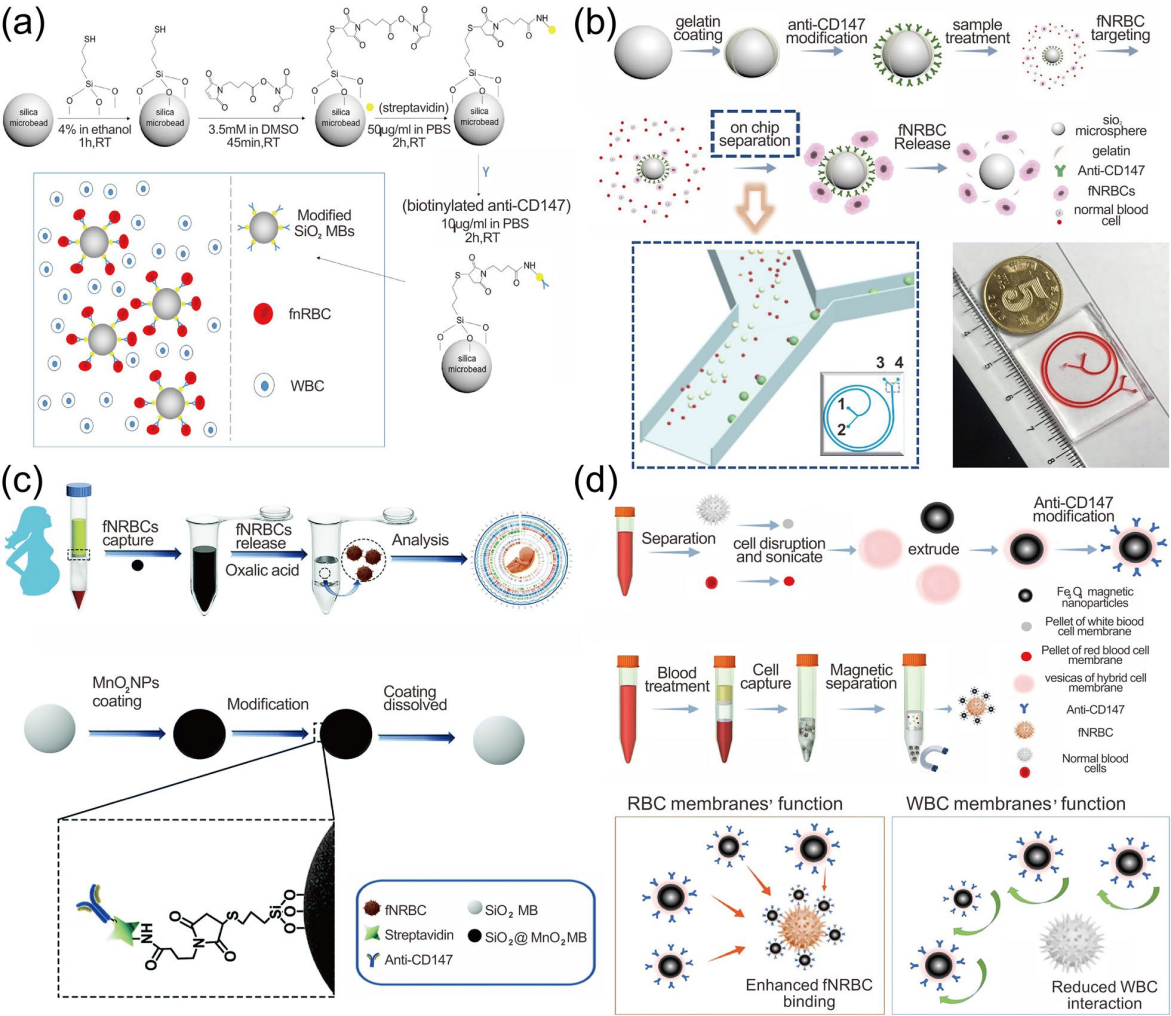
Nanomaterial-enhanced dynamic sorting methods for fNRBC capture. **a** SiO_2_ microbeads modified with SA and anti-CD147 for fNRBC capture. Reprinted with permission from ref [28]. Copyright 2019 John Wiley & Sons. **b** Size-scaled silica spheres based on gelatin nanoparticles encapsulated and modified with anti-CD147 for fNRBC isolation. Reprinted with permission from ref [14]. Copyright 2020 Springer Nature. **c** Self-assembled SiO_2_@MnO_2_ microbeads modified by anti-CD147 for sorting fNRBCs. Reprinted with permission from ref [38]. Copyright 2021 The Royal Society of Chemistry. **d** A magnetic nanoparticle encapsulated with a mixed membrane of leukocytes and erythrocytes to capture fNRBCs. Reprinted with permission from ref [170]. Copyright 2021 American Chemical Society

To increase the concentration of target cells and reduce the adhesion of non-specific cells, researchers have used micro-/nanoscale microspheres to dynamically capture target cells (Fig. 4). In 2020, Cheng et al. [28] used high-density SiO_2_ microbeads to capture fNRBCs for non-invasive ABO blood grouping. The SiO_2_ microbeads were modified with streptavidin and antibodies (Fig. 4a). The origin of the captured cells was detected using anti-HbF-PE, anti-CD71-FITC, and DAPI by trichrome fluorescence analysis. Next, DNA was extracted from the cells and the fetal ABO blood group was detected using PCR. The results showed the feasibility of this method, providing more possibilities for the application of non-invasive detection of fetal ABO blood groups in early pregnancy. The platform allows pregnant women, especially those with blood type O, to know the fetal blood type in early pregnancy, providing reliable support for delivery and postpartum. Unfortunately, this method can’t release the cells well. To improve the efficiency of cell release, in 2020, Wang et al. [14] prepared large-sized silica spheres based on gelatin nanoparticles encapsulated and modified with anti-CD147, followed by microarray filtration to obtain fNRBCs (Fig. 4b). This was immediately followed by the addition of metalloprotease to degrade the gelatin layer, which improved the release efficiency of target cells as well as cell activity. In 2021, Zhang et al. [38] further optimized the scheme and designed a self-assembled MnO_2_ nanoparticle (SiO_2_@MnO_2_) based on silica microbeads, which enabled the isolation and recovery of fNRBCs in early pregnancy. They prepared SiO_2_@MnO_2_, modified by anti-CD147, and used oxalic acid to dissolve the MnO_2_ nanoparticle coating, thereby releasing fNRBCs, and finally used FISH and STR techniques to detect the captured fNRBCs (Fig. 4c). This platform provides a low-cost and easy-to-operate solution for NIPD.

Due to the high number of WBCs in peripheral blood, they will adhere to the substrate even with dynamically capture and may interference with the downstream analysis. To address this problem, an increasing number of studies have verified that cancer cell membranes, erythrocyte membranes, and leukocyte membranes have anti-leukocyte adhesion effects that greatly increase the purity of cells, and thus, the technique has been widely used for sorting rare cells [162, 168, 170, 177, 178]. For example, Wang et al. [170] achieved the isolation and enrichment of fNRBCs in early pregnancy using a magnetic nanoparticle encapsulated with a mixed membrane of leukocytes and erythrocytes (Fig. 4d). They first encapsulated the prepared cell hybrid membrane on magnetic nanoparticles, followed by anti-CD147 modification, and then verification of the origin of the cells by three-fluorescence staining. The results showed that the isolated target cells were obtained with an efficiency of 90% and a purity of 87%. They then collected peripheral blood samples from pregnant women at 11 to 13 weeks for analysis using the platform and found that 11 to 24 fNRBCs were collected per mL of blood. After, the platform successfully detected a series of aneuploidy, demonstrating its feasibility for NIPD. The membrane is highly effective against non-specific cell adhesion and can selectively capture cells, and it can also be encapsulated for drug-targeted treatment of tumor diseases, which demonstrates great potential for future tumor diagnosis as well as NIPD. Although the above methods have confirmed the feasibility of fNRBCs for NIPD, the clinical specimens used in the current experiments by researchers are very few, and a large number of clinical validations are necessary for further clinical applications. Therefore, such methods with better specificity, sensitive, and reliability are of great need for NIPD.

## Clinical applications of fNRBCs

**Fig. 5:**
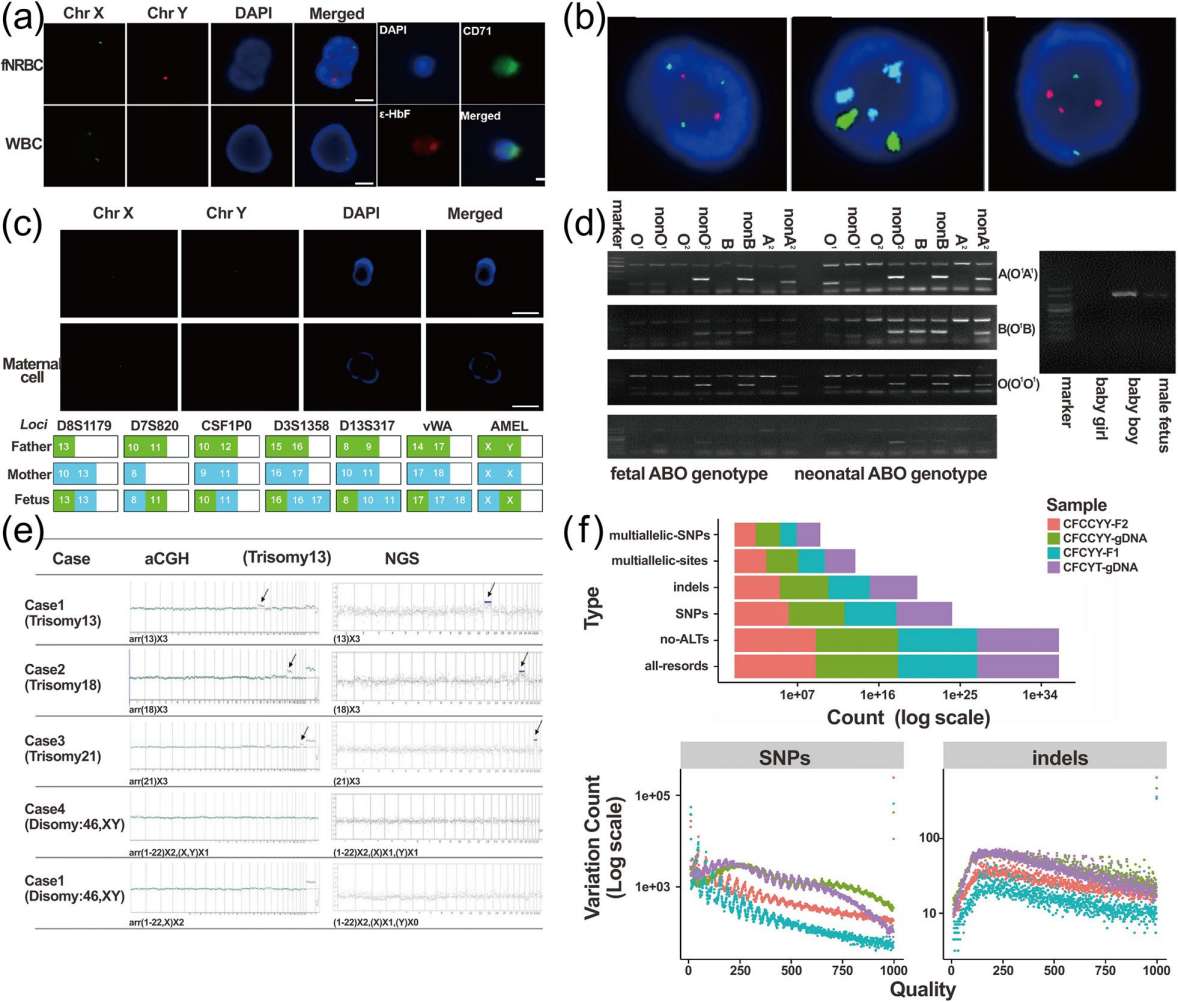
Clinical applications of fNRBCs. **a** Three-color immunofluorescence identification and FISH for detecting the cellular origin of male fetuses. Reprinted with permission from ref [14]. Copyright 2020 Springer Nature **b** FISH for detection of fetal T13, T18, and T21. Reprinted with permission from ref [39]. Copyright 2017 The Author(s). **c** FISH and STR analysis for a female fetus to confirm the cellular origin. Reprinted with permission from ref [38]. Copyright The Royal Society of Chemistry 2021 **d** PCR for detection of fetal blood group. Reprinted with permission from ref [28]. Copyright 2019 John Wiley & Sons. **e** Detection of fetal aneuploidy disorders using aCGH and NGS. Reprinted with permission from ref [39]. Copyright 2017 Springer Nature. **f** WGS for detection of fetal-associated disease variants. Reprinted with permission from ref [222]. Copyright 2017 John Wiley & Sons

Given the rarity of fNRBCs and impurity of the separation, it is necessary to first identify the fNRBCs. The most common method is three-color immunofluorescence identification, which generally uses three different colors of fluorescence to label the membrane and nuclear proteins of fNRBCs, respectively. For example, Wang used PE-labeled anti-ε-globin, FITC-labeled anti-CD71, and DAPI to identify cells, and experiments showed that cells expressing ε-globin+/CD71+/DAPI+were identified as fNRBCs [170]. These fNRBCs need to be released before the downstream identification of the fetal origin and clinical analysis. Common release methods include three major categories: (1) biochemical methods such as DTT [154], MMP-9 [14], oxalic acid solution [38], DNA hybridization [177]; (2) physical methods such as electrical stimulation [30], magnetic field stimulation [170], photo-stimulation [179]; (3) laser microdissection techniques [180]. Since highly specific antibody proteins for fNRBCs have not yet been found, three-immunofluorescence identification makes it difficult to distinguish nucleated erythrocytes from the fetus and mother. If these cells captured by default were all fetal, it would pose a great interference for the analysis of fetal disease downstream. Therefore, it needs to be further identified as to their origin before downstream analysis [11]. Common identification methods include two methods: (1) The probe of FISH for amplifying the SRY gene [35, 170]; (2) STR locus analysis [38]. The identified fNRBCs need to be lysed to obtain DNA, followed by further detection of fetal diseases by FISH, STR, PCR, aCGH, NGS, WGS, WES, and other methods to further detect fetal diseases [7, 32]. Currently, FISH and STR analysis are most commonly used to detect copy number variants (CNVs) in fetal chromosomes, such as Down Syndrome and other aneuploidy disorders [39], while PCR techniques can also detect single gene disorders caused by single nucleotide variants (SNVs) such as sickle cell anemia on this basis [40]. If the specific types (micro-rearrangements, mutations, microdeletions, microduplications, substructures, etc.) and locations of variants on chromosomes are further explored, then these need to be combined with aCGH, NGS, WGA, and WES techniques [34, 42, 43, 181]. These techniques can detect all genetic information of the fetus contained in whole gene or whole exon sequences, which can help genetic counseling of both parents to provide more information about the fetus and provide great support for the application for NIPD (Fig. 5).

### Fluorescence in situ hybridization (FISH)

FISH is the detection of fluorescent signals from fluorescently labeled probes and samples in base complementary pairing at specific sites on the chromosome by fluorescence microscopy, allowing the detection of chromosomal abnormalities. This technique is now commonly used to identify the origin of fNRBCs and to detect aneuploidy disorders of chromosomes [32, 170, 182, 183]. For fetal origin identification, the SRY gene of the cells is generally tested. It is a single-copy gene located on the Y chromosome. Therefore, males express the SRY gene and females do not. Therefore, we can use the FISH method to detect the SRY gene to identify the sex and origin of the cell. If the SRY gene is positive, the cell originates from a male fetus rather than the mother. Several researchers have successfully identified the origin and sex of fNRBCs from known male fetuses using this technique [14, 28, 38, 56, 153, 170]. As early as 1993, one author [35] used both mouse monoclonal antibodies (UCHy) and FISH techniques for immunophenotyping and amplification of Y/X chromosome-specific loci GMGY10 and DXZ1, respectively, to identify the origin of the cells. The results showed that 87% of UCHy-positive male fetal cells had only one Y signal and 68% of UCHy-positive female fetal cells had two X signals. The fetal sex determined by this method was consistent with chromosomal analysis. Although it reduces the contamination of leukocytes, the number of fNRBCs is still very small, which has some impact on the downstream FISH analysis. With the development of technology, more and more works have increased the yield of fNRBCs using microfluidic chips and nanomaterials, and FISH has been widely used to identify fetal origin [14, 38, 153, 170]. In 2021, Xu et al. [153] used S-eDAR chips to capture fNRBCs, followed by FISH amplification on-chip and off-chip. The microarray contained a FISH probe, and the cells of male fetuses showed one green and one red fluorescent dot, and female fetuses showed two green, fluorescent dots so that the sex of the fetus could be distinguished. However, the FISH in-chip method could return false positives due to air bubbles and contamination. The final method described used a microscope to collect individual target cells outside the microarray and used a FISH probe to identify the origin and sex of the cells. The results showed that the SRY locus of the Y chromosome was in red and the DXZ1 locus of the X chromosome was in blue. The average success rate of FISH for 261 single cells was 71.6%. In addition, Zhang [38] and Wang [14] also successfully detected the SRY gene using FISH probes (Fig. 5a). Although this method is simple and can also distinguish the sex of the fetus, it is limited to the identification of male fetuses only. Therefore, its application is limited. To know whether the cells are specifically of fetal or maternal origin, STR analysis is required [184].

In the detection of aneuploidy diseases, the FISH technique has also been widely used [32, 170, 182, 183]. As early as 1999, Zhen [183] performed multiple rounds of FISH hybridization using the Poly-FISH technique for specific loci of 13, 18, 21, and sex chromosomes. The results showed that one X, one Y, two 18, and three 21 signals could be detected at the same location in the same fetal cell of T21. This method allowed multiple rounds of hybridization using multiple FISH probes for the same target cell at the same time, thus analyzing multiple chromosomal abnormalities. This greatly improved the hybridization efficiency of FISH, which also had good reproducibility. Later in 2001, a scholar [182] used the FISH method to verify aneuploidy disorders in the state of placental chimerism. They collected six cases of X chromosome haploid and seven cases of chromosome 18 triploid and obtained fNRBCs by chorionic villus sampling, after which the samples were amplified using specific probes for chromosomes X, Y, and 18. The results showed that five of the six haploid cases contained an X signal and covered 89 to 100% of the cells, and 60 to 100% of the cells in T18 contained three X signals. This study demonstrated that the FISH method could also be used for placental chimeric aneuploidy. Therefore, the use of invasive tests in early pregnancy can then identify the cell karyotype, so that secondary damage to the pregnant woman and fetus can be avoided. Furthermore, in 2017, Huang et al. [39] used the FISH method to amplify specific loci of chromosomes 13, 18, and 21 and successfully detected T13, T18, and T21 from 24 pregnant women, respectively (Fig. 5b). The method is simple, and rapid, and allows multiple color markers. This method does have some limitations: the hybridization efficiency is significantly reduced when the probes are relatively short, and the specific location of chromosomal variants requires NGS technology to detect them [185]^.^ Therefore, this method is more suitable for simpler and faster gene amplification and detection of gene CNVs in the laboratory.

### Short tandem repeat (STR)

STR is a sequence of 1–6 nucleotides and is highly expressed in humans with a high degree of polymorphism. Therefore, analysis of specific STR loci on chromosomes can identify the origin of cells as well as chromosomal aneuploidy diseases [39, 186, 187]. According to Mendel’s law, half of STR loci come from the mother and half from the father. Therefore, it is possible to identify the origin and sex of cells by comparing the class, number, and location of genes at the STR loci of the fetus and both parents [188]. For male fetuses, the specific STR genes on the X chromosome are from the mother. For female fetuses, one is from the mother, and the other one is from the father. When the genotype of the female fetus does not match the mother, we can determine that the genotype is from the fetus. However, when the female fetus genotype is consistent with the mother, it may not be easy to determine and needs to be combined with other loci. Some studies have used STR sequences for NIPD and have successfully identified both the origin of the cells and the sex of the fetus [39, 188]. Huang et al. [39] amplified the STR locus AMEL on the X chromosome of the fNRBCs to identify the origin of the cells and the fetal sex. In addition, Giambona et al. [188] amplified seven STR loci of the X/Y chromosome containing AMXY, HPRT, SRY, DXS8377, DXS1187, DXS6803, DXS6809. The electrophoretic profiles of STR showed that 96 of 159 cells contained a maternal peak and a paternal peak. It also indicated that some of these cells belonged to the fetus. Later, Zhang et al. [38] explored the performance of the technique for the identification of female fetal cells by amplifying D8S1179, D7S820, CSF1P0, D3S1358, D13S317, vWA, and AMEL loci from a female fetus and both of her parents, and the results verified that one of the fetal loci was from the mother and one from the father, confirming the technique in identifying the origin of the cells (Fig. 5c). Compared with FISH, this method is less expensive, more sensitive, faster, and not limited to male fetuses. However, it requires the identification of both parents’ genotypes and is more complicated to perform. Both methods (FISH and STR) can identify the origin of fetal cells as well as the sex of the fetus, each with its advantages and disadvantages. It is the identification method that makes captured fetal cells available for analysis in NIPD.

In addition, some researchers have used this technique to diagnose chromosomal aneuploidy disorders [187, 189, 190]. In 1994, Pertl et al. [189] collected DNA from samples to detect T21 by amplification and fluorescent labeling of the specific STR locus D21S11 on chromosome 21. The results demonstrated that eight amniotic fluid samples showed three STR peaks, and another eight samples had two other peaks with a 2:1 ratio. Subsequent cytogenetic analysis of these samples detected abnormalities confirming that they had Down syndrome, which provided evidence for the use of STR for NIPD. Given the existence of fetuses and mothers with identical allelic loci, there are limitations to a single STR locus. To avoid this problem, in 2001, a scholar used flow cytometry to isolate fNRBCs, followed by the amplification of three STR loci D21S11, D21S1411, and D21S1412 to detect aneuploidy disease [187]. They collected blood samples from seven pregnant women with T21, three of which had a fetal genotype of 47, XY,+21, and the other four had a fetal genotype of 47, XX,+21. The results suggested that five cases containing three fluorescent peaks or two peaks with a 2:1 ratio were confirmed to have fetuses with T21. This illustrates that STR locus analysis can provide a new method for chromosome detection. Next, Yoon [190] simultaneously amplified STR loci on chromosomes 18 and 21 to detect Down and Edwards syndrome. They detected them with abnormal STR peaks by comparing the area ratios of STR peaks in 47 cases of normal karyotype, 23 cases of Down syndrome, and 8 cases of Edwards syndrome. This method allows rapid detection of trisomy within 8 h, so it provides a new protocol for rapid prenatal screening of Down and Edwards syndrome. In addition, Huang et al. [39] detected multiple specific STR loci for chromosomes 13, 18, and 21 and successfully detected trisomies. Therefore, the amplification of multiple STR loci can be used as a complementary technique for the diagnosis of aneuploidy diseases. The advantages of this technique in identifying the cellular fetal origin, fetal sex, and aneuploidy disorders make it widely applicable. It also provides a new potential mechanism for further identification and analysis of target cells.

### Polymerase chain reaction (PCR)

Genetic deletions, rearrangements, or mutations occur in fetal cells and can result in several severe illnesses such as Down Syndrome [191], sickle cell anemia [38, 40], β-thalassemia [40], fetal hemolysis [28], spinal muscular atrophy [192], and Duchenne muscular dystrophy [193], which can all be detected by PCR. Back in 1996, Pertl [194] used quantitative fluorescent PCR (QF-PCR) to amplify the specific sequences D21S11, D21S1414, MBP, and AMXY on chromosomes 18 and 21 to detect T18 and T21. They performed PCR amplification of these specific sequences, followed by electrophoretic analysis using polyacrylamide gels and DNA sequencers, and used specific software to analyze the relative fluorescence intensity of the products. The results showed that 10 of the 20 trimers were three peaks when they were labeled using D21S11 and D21S1414. The other 10 were 2:1 with two peaks. Also, when D21S1414, MBP, and AMXY markers were used simultaneously, the map was a bi-equivalent trisomy pattern. It confirmed the feasibility of QF-PCR to detect trisomies. This method allows rapid detection of trisomies using a smaller number of samples and provides a new tool for NIPD. In the same year, Cheung et al. [40] used MACS and micromanipulation techniques to isolate single fNRBCs and then amplified the extracted DNA to successfully detect sickle cell anemia and thalassemia disease. It showed the feasibility of using PCR methods to diagnose monogenic diseases. In addition, in 2020, Cheng et al. [28] used anti-CD147-modified silica spheres to capture fNRBCs, followed by DNA extraction and amplification of the ABO blood group gene of the cells using the PCR technique (Fig. 5d). They collected peripheral blood from 52 pregnant women, including 26 pregnant women with blood group O and 27 male fetuses. The results showed that the technique could successfully detect the blood type of 26 fetuses and that the genotype of the fetus was the same as the blood type at birth. In addition, they also amplified the SRY gene by PCR, and the results showed that the SRY gene was detected in all 27 male fetuses. Thus, this technique allows both parents to know the fetal blood type in advance, facilitating early screening and prevention of neonatal hemolysis. Since fNRBCs obtained from blood samples often mix with many background cells, downstream analysis is quite challenging. Therefore, this technique generally requires high sample purity in addition to avoiding contamination during amplification.

### Array comparative genomic hybridization (aCGH)

Common aneuploid diseases or monogenic diseases can be detected by FISH, STR, and PCR techniques. However, these techniques have low resolution and require high cell activity and purity. In contrast, the advantages of aCGH, such as high resolution, the release of downed cells without culture, and the ability to detect unbalanced rearrangements of submicroscopic chromosomes, led to its wide use in exploring fetal chromosomal variation [195]. The aCGH technique, also called Gene Chip technology, uses different kinds of fluorescence to label DNA samples of the experimental and the control group, and hybridizes them with specific probes to determine the CNVs of the samples of that experimental group by comparing the differences in fluorescence intensity between them, and then processes and interprets these variant signals with specialized detection software [196]. It can detect any genetic variations and sequence copy number changes without the need to select a specific region in advance and know the validation information of chromosomal abnormalities in that study region. This enables accurate localization and analysis of some specific mutation sites [197] for identifying unknown gene fragments in prenatal diagnosis [191]. Huang et al. [39] performed the aCGH analysis of DNA amplification products from five fNRBCs (one T13, T18, and T21 each in the experimental group and two normal karyotypes in the control group) and the plots showed fetuses from the experimental group with abnormal chromosomal peaks (Fig. 5e). In addition, Le et al. [198] successfully detected 49 chromosomal abnormalities, including polyploid karyotypes and variants of normal karyotypes with submicron structures by using microarrays with this technique. In addition, 8 types of gene rearrangements were detected and confirmed by QF-PCR, demonstrating the potential of the technique in NIPD.

Compared with conventional karyotype analysis, this technique does not require cell culture techniques but instead detects chromosomal abnormalities by directly testing DNA from fetal cells or amniotic fluid samples [199]. Cheung et al. [199] analyzed 2585 samples using the aCGH technique and karyotype analysis, respectively, and the results showed that aCGH technology detected 12 cases of chromosomal chimerism, while the latter only detected 2 cases, further demonstrating the advantages of aCGH technology. The technique can detect chromosomal aneuploidy, microduplications, and microdeletions in unbalanced rearrangements [200]. For some balanced rearrangements, it cannot distinguish well between polyploid rearrangements of the same chromosomes. Because of the imbalance of sex chromosomes, it is easy to detect trisomies in males. Ballif et al. [201] used aCGH microarrays to detect aneuploidy in 11 chromosomal abnormal cell lines. They extracted DNA from the experimental and reference groups and labeled them using Cy3 and Cy5, respectively, and hybridized on one microarray, followed by reverse labeling and then hybridization on another, explaining the chromosomal variation by comparing the difference in the average ratio of the fluorescence intensities of the two. The study found that the difference between male trisomy (47, XXY) and normal males was significant, while the ratio of fluorescence intensity difference between normal women (46, XX) and trisomy (47, XXX) was minimal. Using a normal male cell line as a reference for trisomies, it was found that male trisomy (69, XXY) produced a different characteristic plot from normal males, while female trisomy (69, XXX) produced the same characteristic plot as normal females, demonstrating the excellent sensitivity of the technique in detecting non-equilibrium polyploidy. However, female trisomy cannot be well distinguished from normal diploidy, and the FISH technique needs to be further determined at this time, limiting its clinical application [201, 202].

This technique has greatly improved the resolution and detection rate of chromosomal abnormalities, providing parents with more reliable genetic information [198, 201]. When using this technique, one must avoid contamination of sample DNA to prevent interference with downstream analysis. Furthermore, the increased resolution of chromosomal abnormalities has led to increased uncertainties about the clinical significance of CNVs [203]. It not only increases the workload of DNA analysis but also causes anxiety to parents when some adverse genetic abnormalities are found in infants and children [204]. As a result, more knowledge about the relevant CNVs is needed to better understand and further analyze the clinically unclear CNVs, which provides lots of support for prenatal diagnosis [205].

### Next-generation sequencing (NGS)

NGS is also called high-throughput sequencing. It can sequence hundreds of thousands or even millions of genes at one time. Compared with Conventional sequencing methods, it can also offer a comprehensive analysis of the genomic and transcriptomic information of fetal cells at high throughput and low cost [206–208]. The technique is currently used for the analysis of aneuploidy and CNVs, screening for monogenic diseases, and whole-genome sequencing [19, 43, 209–212]. It is known that Conventional PCR and FISH methods can only analyze simple single-gene abnormalities, while aCGH technology cannot analyze some chromosomal balanced abnormalities due to its resolution limitation. With the rapid development of NGS technology, the above limitations have been broken. It was earlier used for pre-implantation genetic analysis to accurately screen and evaluate abnormal embryos, thereby discarding abnormal embryos and selecting normal embryo implantation, which provided a reduction in infant birth defects and improved quality of life [213]. Later researchers used NGS technology to detect trophoblast cells [214], cffDNA [215], and fNRBCs [209] for the analysis of fetal aneuploidy and SNVs. Hua et al. [212] used NGS techniques to detect the aneuploidy of fNRBCs by taking 4 cell samples of known aneuploidy and using Illumina MiSeq for sequencing. The results showed that T21 and T18, using the 0.08×sequencing depth, were able to accurately detect each fNRBCs in these four cases.

Furthermore, NGS can also be used to detect and evaluate the CNVs of chromosomes in fetuses including trisomy and microdeletion/microduplication syndromes (MMS) [42, 210]. Liang et al. [42] collected 1128 cases of pregnancy abnormalities, including 965 cases of aneuploidy and 163 cases of MMS. For common trisomies like T21 and T18, the positive predictive values (PPVs) were 95% and 82%, respectively. Among the 120 MMS cases explored, 32 were associated with common chromosomal disorders, including DiGeorge syndrome (DGS), 22q microduplication syndrome, Prader-Willi/Angleman syndrome (PWS), and Cri du Chat (CDC) with PPVs of 93%, 68%, 75%, and 50%, respectively. For the remaining 88 cases, they could not identify chromosomal changes from the current gene pool, and their PPVs were 32% (CNVs $\ge$ 10 Mb) and 19% (CNVs < 10 Mb), respectively. The results demonstrated that the non-invasive prenatal screening platform produced higher PPVs on aneuploidy and DGS, with higher sensitivity and specificity. However, the presence of placental mosaicism makes the PPVs of some other CNVs lower. Therefore, the platform can be used in combination with ultrasound as an initial screening tool. In addition, NGS technology has been used to detect monogenic diseases such as congenital deafness and ichthyosis [41], and thalassemia [211]. Chang et al. [41] established a noninvasive diagnostic method for isolating fetal cells and detecting single-gene diseases. They first collected peripheral blood from pregnant women with congenital deafness and ichthyosis. After identifying and picking up individual fetal cells by an automated high-throughput image analysis system, they identified the cell source and analyzed it using NGS technology and SNP haplotype methods. The results showed that the sequencing results of fetal cells of these two diseases were consistent with the amniotic fluid testing. The method was shown to be advantageous in detecting single-gene genetic abnormalities and assessing fetal health, although more clinical data are needed to further evaluate the performance of the method. Compared with aCGH technology, NGS technology has higher sensitivity and can detect the balance rearrangement of chromosomes [216]. This strategy allows the accurate identification of chromosomal rearrangements and reduces the workload and cost to a great extent.

The method allows high-throughput comprehensive analysis of the genome and transcriptome of a species. It can be used for single or multiple mutation analysis simultaneously. This indicates that it can sequence hundreds of millions of small gene fragments at the same time, which greatly improves the efficiency of disease detection. However, the technique is costly and cannot analyze single target cells well, and a further combination of WGS and WES is required for single-cell analysis [7].In addition, some CNVs generated during the experiment cannot yet be systematically interpreted using the current clinical CNVs database, so the use of NGS technology can generate some variants of unknown clinical significance, which poses a great challenge for the genetic counseling of patients [210].

### Whole-genome sequencing (WGS)

With the continuous development of high-throughput sequencing technology, WGS has been widely used for single-gene genetic diseases, cancer, and complex gene detection due to its decreasing cost and continually maturing analysis system; it has become an important tool for clinical detection of genetic variants [217–219]. The technique can detect all gene sequences of the fetal cells, thus finding SNVs, insertions or microdeletions (InDels), CNVs, and structural variants (SVs) within coding and non-coding regions, which provides comprehensive clinical information on mutations. It can currently identify the origin of fNRBCs [34], and detect aneuploidy of fNRBCs [39, 212, 220]. Compared with conventional PCR, FISH, and karyotype analysis, WGS can detect all genetic variants within a single cell in high throughput. Therefore, the technique can be used to detect variants that cannot be detected by conventional sequencing methods, which reduces the error rate caused by the deletion of genes due to conventional PCR methods [221]. For example, Chen et al. [222] used the WGS method to analyze the sequences obtained by high-coverage amplification and to detect variants in fetal cells to assess monogenic genetic disorders, followed by deep sequencing of genomic DNA (gDNA) from the same newborns to provide validation of these variants (**Fig. ****5****f**). The results indicated cellular coverage and allelic deletion rates of 86.8% and 24.90%, respectively. In addition, after single-gene genetic disease analysis, they identified a total of 5344 variants, which were all confirmed by deep gDNA analysis in newborns. It illustrates that WGS can provide more comprehensive genetic information for NIPD. WGS scans the entire gene sequence to find mutation sites associated with the related disease, and studies have shown that more than 800 mutation sites can be found in a single sequencing [206]. However, some variants are from non-coding regions and these genes are not well studied yet, so they cannot be explained by current clinical knowledge. It was shown that the general population also carried some variants that were not expressed and not pathogenic in the initial examination [223]. Therefore, we need to further differentiate and determine whether these variants are pathogenic as well as epiphenomenal, increasing the workload and cost to some extent. In addition, considering that ultrasound can only generally detect changes in fetal structure beyond 20 weeks of pregnancy, physicians need to detect and reassess these variants as soon as possible when the fetus shows abnormalities. In conclusion, this technique may be used as an initial screening method for NIPD. The detected genetic variants can be referenced for prenatal diagnosis and can help physicians further determine the pregnant woman's risk level. The risk of unnecessary invasion can be avoided for low-risk pregnancies. For high-risk pregnancies, further methods such as family genetic history and genetic testing for specific diseases can be combined to detect fetal abnormalities. In addition, WGS also provides more comprehensive fetal genetic information for both parents, which can be used as a reference for phenotypic analysis of the fetus after birth. However, studies have found that not all fetal phenotypes are consistent with neonatal phenotypes. This poses a great challenge for the application of WGS. Therefore, researchers are needed to further understand as well as refine the performance of this method and to choose a more appropriate method according to the specific situation of the pregnant woman to avoid the increase of potential risks [19].

### Whole exome sequencing (WES)

WGS is costly, has complex equipment, and requires staff with a sophisticated bioinformatics background, so its clinical application is greatly limited [217]. Exonic regions represent 1% of the whole genome but occupy more than 85% of the genes associated with diseases. This is coupled with the low cost of the technique and the small sequencing range. Therefore, sequencing and evaluation of exonic sequences of fNRBCs have become a popular sequencing modality today [30, 224, 225]. For example, Feng et al. [30] obtained fNRBCs from high-risk fetuses by polypyrrole nanoparticle microarrays and explored aneuploidy and microdeletion variants of fetal chromosomes using WES. To facilitate observation, they used three colors of fluorescent probes to detect aneuploidy disorders in fetuses. As a result, 12 aneuploidies were detected (including 5 cases of T21, 2 cases of T18, 3 cases of T13, and 2 cases of Klinefelter’s syndrome), and the accuracy of the technique was then confirmed using amniocentesis and G-band karyotyping. In addition, they identified the 18q21 microdeletion of the DYM gene in the case of fetal cells in which ultrasound showed abnormal organ architecture. This illustrates the potential of the WES technique in detecting variants such as chromosome copy numbers and microdeletions. We know that not all mutations are inherited, and even if a full genetic test is performed on a fetus before birth, children with normal test results may be found to have abnormalities because of spontaneous mutations later in life, so at this time, the choice of low-cost WES technology that can detect more than 85% of diseases in a smaller area is certainly a more comprehensive consideration. To better understand the differences between WGS and WES technologies, Belkadia et al. [226] collected six unrelated genomes and explored the performance of the two technologies. The data showed that for the detection of SNVs and InDels, WES detected an average of 84,192 and 13,325, and WGS detected an average of 84,968 and 12,702. They then analyzed various parameters of both variants and found that WGS could detect 656 high-quality SNVs, while WES could only detect 105, and the rest of the SNVs were thus missed by WES. However, the false-positive variation rates of InDels were similar for both. Overall, the detection of SNVs using WES is not very reliable compared to detection using WGS. Although the cost of WGS is currently high, it shows great advantages in single-gene inheritance. In addition, considering that the specificity of current methods for capturing cells is not high, the actual base sequences that need to be sequenced are much more than expected. When WES is chosen, it also increases substantial costs. Therefore, WGS technology is still the preferred method for detecting genetic variants. In the future, as the cost of WGS technology decreases and bioinformatics analysis capability improves, its ability to detect genetic variants will also improve greatly. However, low-coverage WGS technology will miss many variants, so a high-coverage WGS sequencing platform is our more desirable sequencing method at present [224]. In conclusion, conventional FISH and karyotype analysis can only detect aneuploidy disorders caused by chromosomal variants as well as structural and mechanical aberrations. The aCGH technology can further detect chromosomal abnormalities caused by microduplications, microdeletions, micro-rearrangements, and substructures. However, SNVs, InDels, and other variants need to be analyzed in combination with NGS, WGS, and WES. For clinical use, the physician needs to choose a specific analysis technique for a particular population.

## Summary and outlook

The fNRBCs are promising biomarkers for NIPD, which can provide entire genetic information of the fetus and allow early screening and diagnosis of fetal diseases by simple sampling of maternal blood. Since fNRBCs are rare cells in maternal blood, effective isolation, and detection fNRBCs are crucial for NIPD. Although different methods have been developed for improving the separation efficiency and purity of fNRBCs, challenges remain in getting fNRBCs with high-purity, -yield, and -quality for downstream analysis. (1) Lack of highly specific antibodies: More specific antibodies are needed to enrich fNRBCs, although Anti-CD71, anti-GPA, and anti-CD147 are commonly used for improving the purity of cells. Since anti-CD71 and anti-GPA are also expressed in early maternal NRBCs, more specific antibodies are of great need. Another possibility is the exploitation of microfluidics and physical cell separation based on the unique properties of fNRBCs by reducing the adhesion of non-specific cells. (2) Difficulty to identify the origin of the cells. Currently, FISH technique and STR analysis are commonly used to identify fNRBCs. However, the FISH technique can only be used to identify male fNRBCs and has limited application, while the STR analysis is complicated to operate since it requires the combination of multiple STR sites and a comparison of fetal and bi-parental genes. Therefore, advanced methods are needed to identify the origin of cells. (3) Lack of good methods for clinical fNRBC separation. The micro/nanotechnologies advance rare cell isolation based on their advantages of high throughput, integration, and miniaturization. Through the integration of nanostructures, affinity surfaces, and microfluidic operations, and these novel approaches offer more possibilities for the better and faster capture and high-throughput analysis of fNRBCs. However, NIPD methods based on these technologies are still not translated or commercialized for widely clinic usages yet. (4) Limited translational applications: The current NIPD technology is still in its infancy. Although the technology has been approved to analyze single-gene diseases, diagnose aneuploidy, and identify blood groups, the actual detection of fetal disease is rare. There are still technique barriers that limit the clinical application of NIPD targeting fNRBCs. To overcome above changes, the further research efforts may be made for several aspects: (1) it could be promising to find biomarkers with higher specificity to obtain high purity fNRBCs through the in-depth analysis of the molecules that express fNRBCs and the microenvironment in which they live; (2) attempts may be made to find a lower cost, simpler operation, and more comprehensive analysis method for fetal origin identification; (3) The NIPD technology calls for fNRBC isolation/separation methods that are low cost, simple to operate, and can be industrialized for massive production; (4) New technologies and automated engineering systems are required for the comprehensive detection and analysis of individual fNRBCs and non-invasive prenatal clinical applications to achieve early detection, early diagnosis, early treatment, and early intervention of fetal diseases.

## Data Availability

The dataset supporting this review article is included within all the cited articles.

## Abbreviations

NIPD: Noninvasive prenatal diagnosis
fNRBCs: Fetal nucleated red blood cells
RBCs: Red blood cells
WBCs: White blood cells
cffDNA: Cell-free fetal DNA
GPA: Glycophorin A
HbF: Hemoglobin F
HbA: Hemoglobin A
SBA: Soybean agglutinin
DGC: Density gradient centrifugation
MACS: Magnetic-activated cell sorting
FACS: Fluorescence-activated cell sorting
DLD: Deterministic lateral displacement
DEP: Dielectrophoresis
FISH: Fluorescence in situ hybridization
PCR: Polymerase chain reaction
STR: Short tandem repeat
aCGH: Array comparative genomic hybridization
NGS: Next-generation sequencing
WGA: Whole genome amplification
WGS: Whole-genome sequencing
WES: Whole exome sequencing
SNVs: Single nucleotide variants
CNVs: Copy number variants

## References

[1] Groisman B, Bermejo-Sánchez E, Romitti PA, Botto LD, Feldkamp ML, Walani SR, Mastroiacovo P (2019). Join world birth defects day. Pediatr Res.

[2] Czeizel AE, Gasztonyi Z, Kuliev A (2005). Periconceptional clinics: a medical health care infrastructure of new genetics. Fetal Diagn Ther.

[3] Gonzaludo N, Belmont JW, Gainullin VG, Taft JR (2019). Estimating the burden and economic impact of pediatric geneticdisease. Genet Med.

[4] Cheng WL, Hsiao CH, Tseng HW, Lee TP (2015). Noninvasive prenatal diagnosis. Taiwan J Obstet Gynecol.

[5] Jauniaux E, Rodeck C (1995). Use, risks and complications of amniocentesis and chorionic villous sampling for prenatal diagnosis in early pregnancy. Early Pregnancy.

[6] Tabor A, Alfirevic Z (2010). Update on procedure-related risks for prenatal diagnosis techniques. Fetal Diagn Ther.

[7] Scotchman E, Chandler N, Mellis R, Chitty L (2020). Noninvasive prenatal diagnosis of single-gene diseases: the next frontier. Clin Chem.

[8] Seror V, Muller F, Moatti JP, Le Gales C, Boue A (1993). Economic assessment of maternal serum screening for Down's syndrome using human chorionic gonadotropin. Prenat Diagn.

[9] Torrents-Barrena J, Piella G, Masoller N, Gratacos E, Eixarch E, Ceresa M, Ballester MAG (2019). Segmentation and classification in MRI and US fetal imaging: Recent trends and future prospects. Med Image Anal.

[10] Minear MA, Lewis C, Pradhan S, Chandrasekharan S (2015). Global perspectives on clinical adoption of NIPT. Prenat Diagn.

[11] Mavrou A, Kouvidi E, Antsaklis A, Souka A, Kolialexi A (2010). Identification of nucleated red blood cells in maternal circulation: a second step in screening for fetal aneuploidies and pregnancy complications. Prenat Diagn.

[12] Uitto J, Pfendner E, Jackson LG (2003). Probing the fetal genome: progress in non-invasive prenatal diagnosis. Trends Mol Med.

[13] Hatt L, Brinch M, Singh R, Moller K, Lauridsen RH, Schlutter JM, Uldbjerg N, Christensen B, Kolvraa S (2014). A new marker set that identifies fetal cells in maternal circulation with high specificity. Prenat Diagn.

[14] Wang Z, Cheng L, Wei X, Cai B, Sun Y, Zhang Y, Liao L, Zhao XZ (2020). High-throughput isolation of fetal nucleated red blood cells by multifunctional microsphere-assisted inertial microfluidics. Biomed Microdevices.

[15] Rabinowitz T, Polsky A, Golan D, Danilevsky A, Shapira G, Raff C, Basel-Salmon L, Matar RT, Shomron N (2019). Bayesian-based noninvasive prenatal diagnosis of single-gene disorders. Genome Res.

[16] Dennis Lo YM, Chiu RWK (2008). Noninvasive prenatal diagnosis of fetal chromosomal aneuploidies by maternal plasma nucleic acid analysis. Clin Chem.

[17] Norton ME, Jacobsson B, Swamy GK, Laurent LC, Ranzini AC, Brar H, Tomlinson MW, Pereira L, Spitz JL, Hollemon D (2015). Cell-free DNA analysis for noninvasive examination of trisomy. N Engl J Med.

[18] Kinnings SL, Geis JA, Almasri E, Wang H, Guan X, McCullough RM, Bombard AT, Saldivar JS, Oeth P, Deciu C (2015). Factors affecting levels of circulating cell-free fetal DNA in maternal plasma and their implications for noninvasive prenatal testing. Prenat Diagn.

[19] Rezaei M, Winter M, Zander-Fox D, Whitehead C, Liebelt J, Warkiani ME, Hardy T, Thierry B (2019). A reappraisal of circulating fetal cell noninvasive prenatal testing. Trends Biotechnol.

[20] Alberry M, Maddocks D, Jones M, Abdel Hadi M, Abdel-Fattah S, Avent N, Soothill PW (2007). Free fetal DNA in maternal plasma in anembryonic pregnancies: confirmation that the origin is the trophoblast. Prenat Diagn.

[21] Pin-Jung C, Pai-Chi T, Zhu Y, Jen Jan Y, Smalley M, Afshar Y, Li-Ching C, Pisarska MD, Hsian-Rong T (2019). Noninvasive prenatal diagnostics: recent developments using circulating fetal nucleated cells. Curr Obstet Gynecol Rep.

[22] Schmorl G: *Pathologisch-anatomische untersuchungen über puerperal-eklampsie.* Vogel; 1893.

[23] Simpson JL, Elias S (1993). Isolating fetal cells from maternal blood - advances in prenatal-diagnosis through molecular technology. JAMA-J Am Med Assoc.

[24] Choolani M, Mahyuddin AP, Hahn S (2012). The promise of fetal cells in maternal blood. Best Pract Res Clin Obstet Gynaecol.

[25] Bianchi DW, Flint AF, Pizzimenti MF, Knoll JH, Latt SA (1990). Isolation of fetal DNA from nucleated erythrocytes in maternal blood. Proc Natl Acad Sci U S A.

[26] Bianchi DW, Zickwolf GK, Yih MC, Flint AF, Geifman OH, Erikson MS, Williams JM (1993). Erythroid-specific antibodies enhance detection of fetal nucleated erythrocytes in maternal blood. Prenat Diagn.

[27] Hamada H, Arinami T, Kubo T, Hamaguchi H, Iwasaki H (1993). Fetal nucleated cells in maternal peripheral blood: frequency and relationship to gestational age. Hum Genet.

[28] Cheng L, Wei X, Wang Z, Feng C, Gong Q, Fu Y, Zhao X, Zhang Y (2020). Silica microbeads capture fetal nucleated red blood cells for noninvasive prenatal testing of fetal ABO genotype. Electrophoresis.

[29] Kuo PL (1998). Frequencies of fetal nucleated red blood cells in maternal blood during different stages of gestation. Fetal Diagn Ther.

[30] Feng C, He Z, Cai B, Peng J, Song J, Yu X, Sun Y, Yuan J, Zhao X, Zhang Y (2018). Non-invasive prenatal diagnosis of chromosomal aneuploidies and microdeletion syndrome using fetal nucleated red blood cells isolated by nanostructure microchips. Theranostics.

[31] Hermansen MC (2001). Nucleated red blood cells in the fetus and newborn. Arch Dis Child Fetal Neonatal Ed.

[32] Tang Y, Tang Q, Luo H, Zhang X, Chen Q, Tang W, Wang T, Yang L, Liao H (2022). Research progress in isolation and enrichment of fetal cells from maternal blood. J Chem.

[33] Jeon YJ, Kwon KH, Kim JW, Pang MG, Jung SC, Kim YJ (2010). Comparision in the yield of fetal nucleated red blood cell between the first-and second-trimester using double density gradient centrifugation. Korean J Obstet Gynecol.

[34] Ito N, Tsukamoto K, Taniguchi K, Takahashi K, Okamoto A, Aoki H, Otera-Takahashi Y, Kitagawa M, Ogata-Kawata H, Morita H (2021). Isolation and characterization of fetal nucleated red blood cells from maternal blood as a target for single cell sequencing-based non-invasive genetic testing. Reprod Med Biol.

[35] Zheng YL, Carter NP, Price CM, Colman SM, Milton PJ, Hackett GA, Greaves MF, Ferguson-Smith MA (1993). Prenatal diagnosis from maternal blood: simultaneous immunophenotyping and FISH of fetal nucleated erythrocytes isolated by negative magnetic cell sorting. J Med Genet.

[36] Gedanken A (2004). Using sonochemistry for the fabrication of nanomaterials. Ultrason Sonochem.

[37] Yue S, Naiqi L, Bo C, Xiaoyun W, Zixiang W, Heng C, Dongshan Z, Yuanzhen Z, Xing-Zhong Z (2020). A biocompatible nanofibers-based microchip for isolation and nondestructive release of fetal nucleated red blood cells. Adv Mater Interfaces.

[38] Zhang Q, Zhang K, Guo Y, Wei X, Sun Y, Cai B, Shi Y, Du Y, Liu Y, Fan C, Zhao XZ (2021). The isolation and analysis of fetal nucleated red blood cells using multifunctional microbeads with a nanostructured coating toward early noninvasive prenatal diagnostics. J Mater Chem B.

[39] Huang CE, Ma GC, Jou HJ, Lin WH, Lee DJ, Lin YS, Ginsberg NA, Chen HF, Chang FM, Chen M (2017). Noninvasive prenatal diagnosis of fetal aneuploidy by circulating fetal nucleated red blood cells and extravillous trophoblasts using silicon-based nanostructured microfluidics. Mol Cytogenet.

[40] Cheung MC, Goldberg JD, Kan YW (1996). Prenatal diagnosis of sickle cell anaemia and thalassaemia by analysis of fetal cells in maternal blood. Nat Genet.

[41] Chang L, Zhu X, Li R, Wu H, Chen W, Chen J, Liu H, Li S, Liu P (2021). A novel method for noninvasive diagnosis of monogenic diseases from circulating fetal cells. Prenat Diagn.

[42] Liang D, Cram DS, Tan H, Linpeng S, Liu Y, Sun H, Zhang Y, Tian F, Zhu H, Xu M (2019). Clinical utility of noninvasive prenatal screening for expanded chromosome disease syndromes. Genet Med.

[43] Beaudet AL (2016). Using fetal cells for prenatal diagnosis: History and recent progress. Am J Med Genet C Semin Med Genet.

[44] Kitagawa M, Sugiura K, Omi H, Akiyama Y, Kanayama K, Shinya M, Tanaka T, Yura H, Sago H (2002). New technique using galactose-specific lectin for isolation of fetal cells from maternal blood. Prenat Diagn.

[45] Sekizawa A, Watanabe A, Kimura T, Saito H, Yanaihara T, Sato T (1996). Prenatal diagnosis of the fetal RHD blood type using a single fetal nucleated erythrocyte from maternal blood. Obstet Gynecol.

[46] Choolani M, O'Donoghue K, Talbert D, Kumar S, Roberts I, Letsky E, Bennett PR, Fisk NM (2003). Characterization of first trimester fetal erythroblasts for non-invasive prenatal diagnosis. Mol Hum Reprod.

[47] Samura O, Sekizawa A, Zhen DK, Falco VM, Bianchi DW (2000). Comparison of fetal cell recovery from maternal blood using a high density gradient for the initial separation step: 1.090 versus 1.119 g/ml. Prenatal Diagn.

[48] Ganshirt-Ahlert D, Borjesson-Stoll R, Burschyk M, Dohr A, Garritsen HS, Helmer E, Miny P, Velasco M, Walde C, Patterson D (1993). Detection of fetal trisomies 21 and 18 from maternal blood using triple gradient and magnetic cell sorting. Am J Reprod Immunol.

[49] Kwon KH, Jeon YJ, Hwang HS, Lee KA, Kim YJ, Chung HW, Pang MG (2007). A high yield of fetal nucleated red blood cells isolated using optimal osmolality and a double-density gradient system. Prenat Diagn.

[50] Simard C, Cloutier M, Jobin C, Dion J, Fournier D, Neron S (2016). Implementing a routine flow cytometry assay for nucleated red blood cell counts in cord blood units. Int J Lab Hematol.

[51] Houyhongthong V, Nunphuak W, Sripatumtong C, Parnsamut C, Ketloy C (2018). Automated nucleated red blood cell count using the Mindray BC-6800 hematology analyzer. Int J Lab Hematol.

[52] Bohmer RM, Zhen D, Bianchi DW (1998). Differential development of fetal and adult haemoglobin profiles in colony culture: isolation of fetal nucleated red cells by two-colour fluorescence labelling. Br J Haematol.

[53] Yurtcu E, Karcaaltincaba D, Kazan HH, Ozdemir H, Yirmibes Karaoguz M, Calis P, Kayhan G, Guntekin Ergun S, Percin F, Bayram M (2021). Is cervical swab an efficient method for developing a new noninvasive prenatal diagnostic test for numerical and structural chromosome anomalies?. Turk J Med Sci.

[54] Zheng S, Tong X, Wu L, He G, Ding B, Yao L, Liu Y (2012). A comparison of in vitro culture of fetal nucleated erythroblasts from fetal chorionic villi and maternal peripheral blood for noninvasive prenatal diagnosis. Fetal Diagn Ther.

[55] Fukushima A, Utsugisawa Y, Wada Y, Mizusawa N, Horiuchi S, Kagabu T (2001). The application of magnetic cell sorter (MACS) to detect fetal cells in maternal peripheral blood. J Obstet Gynaecol Re.

[56] Nemescu D, Constantinescu D, Gorduza V, Carauleanu A, Dan BN (2020). Comparison between paramagnetic and CD71 magnetic activated cell sorting of fetal nucleated red blood cells from the maternal blood. J Clin Lab Anal.

[57] Babochkina T, Mergenthaler S, Lapaire O, Kiefer V, Yura H, Koike K, Holzgreve W, Hahn S (2005). Evaluation of a soybean lectin-based method for the enrichment of erythroblasts. J Histochem Cytochem.

[58] Kanda E, Yura H, Kitagawa M (2016). Practicability of prenatal testing using lectin-based enrichment of fetal erythroblasts. J Obstet Gynaecol Res.

[59] Takabayashi H, Kuwabara S, Ukita T, Ikawa K, Yamafuji K, Igarashi T (1995). Development of non-invasive fetal DNA diagnosis from maternal blood. Prenat Diagn.

[60] Giambona A, Damiani G, Leto F, Jakil C, Renda D, Cigna V, Schillaci G, Picciotto F, Nicolaides KH, Passarello C (2016). Embryo-fetal erythroid cell selection from celomic fluid allows earlier prenatal diagnosis of hemoglobinopathies. Prenat Diagn.

[61] Sekizawa A, Kimura T, Sasaki M, Nakamura S, Kobayashi R, Sato T (1996). Prenatal diagnosis of Duchenne muscular dystrophy using a single fetal nucleated erythrocyte in maternal blood. Neurology.

[62] Nagy GR, Ban Z, Sipos F, Beke A, Papp C, Papp Z (2005). Isolation of epsilon-haemoglobin-chain positive fetal cells with micromanipulation for prenatal diagnosis. Prenat Diagn.

[63] Oosterwijk JC, Knepflé CF, Mesker WE, Vrolijk H, Sloos WC, Pattenier H, Ravkin I, van Ommen G-JB, Kanhai HH, Tanke HJ (1998). Strategies for rare-event detection: an approach for automated fetal cell detection in maternal blood. Am J Human Genet.

[64] Wei X, Chen K, Guo S, Liu W, Zhao XZ (2021). Emerging microfluidic technologies for the detection of circulating tumor cells and fetal nucleated red blood cells. ACS Appl Bio Mater.

[65] Li R, Zhang X, Lv X, Geng L, Li Y, Qin K, Deng Y (2017). Microvalve controlled multi-functional microfluidic chip for divisional cell co-culture. Anal Biochem.

[66] Autebert J, Coudert B, Bidard FC, Pierga JY, Descroix S, Malaquin L, Viovy JL (2012). Microfluidic: an innovative tool for efficient cell sorting. Methods.

[67] Chan CY, Huang P-H, Guo F, Ding X, Kapur V, Mai JD, Yuen PK, Huang TJ (2013). Accelerating drug discovery via organs-on-chips. Lab Chip.

[68] Deng B, Tia Y, Yu X, Song J, Guo F, Xiao Y, Zhang Z (2014). Laminar flow mediated continuous single-cell analysis on a novel poly(dimethylsiloxane) microfluidic chip. Anal Chim Acta.

[69] Guo F, French JB, Li P, Zhao H, Chan CY, Fick JR, Benkovic SJ, Huang TJ (2013). Probing cell-cell communication with microfluidic devices. Lab Chip.

[70] Zhao Y, Stratton ZS, Guo F, Lapsley MI, Chan CY, Lin S-CS, Huang TJ (2013). Optofluidic imaging: now and beyond. Lab Chip.

[71] Guo F, Li S, Caglar MU, Mao Z, Liu W, Woodman A, Arnold JJ, Wilke CO, Huang TJ, Cameron CE (2017). Single-cell virology: on-chip investigation of viral infection dynamics. Cell Rep.

[72] Liu H, Wang Y, Cui K, Guo Y, Zhang X, Qin J (2019). Advances in hydrogels in organoids and organs-on-a-chip. Adv Mater.

[73] Lin DSY, Guo F, Zhang B (2019). Modeling organ-specific vasculature with organ-on-a-chip devices. Nanotechnology.

[74] Ao Z, Song S, Tian C, Cai H, Li X, Miao Y, Wu Z, Krzesniak J, Ning B, Gu M (2022). Understanding immune-driven brain aging by human brain organoid microphysiological analysis platform. Adv Sci (Weinh).

[75] Ao Z, Cai H, Wu Z, Hu L, Li X, Kaurich C, Gu M, Cheng L, Lu X, Guo F (2022). Evaluation of cancer immunotherapy using mini-tumor chips. Theranostics.

[76] Cai H, Ao Z, Tian C, Wu Z, Kaurich C, Chen Z, Gu M, Hohmann AG, Mackie K, Guo F (2023). Engineering human spinal microphysiological systems to model opioid-induced tolerance. Bioact Mater.

[77] Ao Z, Cai H, Wu Z, Hu L, Nunez A, Zhou Z, Liu H, Bondesson M, Lu X, Lu X (2022). Microfluidics guided by deep learning for cancer immunotherapy screening. Proc Natl Acad Sci U S A.

[78] Guevara-Pantoja PE, Jimenez-Valdes RJ, Garcia-Cordero JL, Caballero-Robledo GA (2018). Pressure-actuated monolithic acrylic microfluidic valves and pumps. Lab Chip.

[79] Huang M, Zheng L, Zhang H, Xue S, Ni H. Application of microvalve based on computer control in biological chemical and medical. In the 2019 International Conference. Association for Computing Machinery; 2019: 1-6

[80] Illath K, Kar S, Gupta P, Shinde A, Wankhar S, Tseng F, Lim K, Nagai M, Santra T (2022). Microfluidic nanomaterials: from synthesis to biomedical applications. Biomaterials.

[81] Yu ZT, Aw Yong KM, Fu J (2014). Microfluidic blood cell sorting: now and beyond. Small.

[82] Byeon Y, Ki CS, Han KH (2015). Isolation of nucleated red blood cells in maternal blood for non-invasive prenatal diagnosis. Biomed Microdevices.

[83] Mohamed H, Turner JN, Caggana M (2007). Biochip for separating fetal cells from maternal circulation. J Chromatogr A.

[84] Shen Y, Yalikun Y, Tanaka Y (2019). Recent advances in microfluidic cell sorting systems. Sensor Actuat B-Chem.

[85] Lee D, Sukumar P, Mahyuddin A, Choolani M, Xu GL (2010). Separation of model mixtures of epsilon-globin positive fetal nucleated red blood cells and anucleate erythrocytes using a microfluidic device. J Chromatogr A.

[86] Sethu P, Sin A, Toner M (2006). Microfluidic diffusive filter for apheresis (leukapheresis). Lab Chip.

[87] Ji HM, Samper V, Chen Y, Heng CK, Lim TM, Yobas L (2008). Silicon-based microfilters for whole blood cell separation. Biomed Microdevices.

[88] Xu GL, Chan MB, Yang C, Sukumar P, Choolani M, Ying JY (2006). Design and fabrication a microfluidic device for fetal cells dielectrophoretic properties characterization. Int Mems Conf.

[89] Blom MT, Chmela E, Oosterbroek RE, Tijssen R, van den Berg A (2003). On-chip hydrodynamic chromatography separation and detection of nanoparticles and biomolecules. Anal Chem.

[90] Huang LR, Cox EC, Austin RH, Sturm JC (2004). Continuous particle separation through deterministic lateral displacement. Science.

[91] Huang R, Barber TA, Schmidt MA, Tompkins RG, Toner M, Bianchi DW, Kapur R, Flejter WL (2008). A microfluidics approach for the isolation of nucleated red blood cells (NRBCs) from the peripheral blood of pregnant women. Prenat Diagn.

[92] Medoro G, Manaresi N, Leonardi A, Altomare L, Tartagni M, Guerrieri R (2003). A lab-on-a-chip for cell detection and manipulation. IEEE Sens J.

[93] Borgatti M, Altomare L, Abonnec M, Fabbri E, Manaresi N, Medoro G, Romani A, Tartagni M, Nastruzzi C, Di Croce S (2005). Dielectrophoresis-based 'Lab-on-a-chip' devices for programmable binding of microspheres to target cells. Int J Oncol.

[94] Yu CH, Wang H, Wang Y, Cui NX, Zhao X, Rong L, Yi ZC (2017). Protease sensitivity and redistribution of CD71 and glycophorin A on K562 cells. Cell Mol Biol (Noisy-le-grand).

[95] Ding X, Li P, Lin S-CS, Stratton ZS, Nama N, Guo F, Slotcavage D, Mao X, Shi J, Costanzo F, Huang TJ (2013). Surface acoustic wave microfluidics. Lab Chip.

[96] Ozcelik A, Rufo J, Guo F, Gu Y, Li P, Lata J, Huang TJ (2018). Acoustic tweezers for the life sciences. Nat Methods.

[97] Yue W, Zheng A, Bin C, Maram M, Maria B, Xiongbin L, Feng G (2018). Acoustic assembly of cell spheroids in disposable capillaries. Nanotechnology.

[98] Zhang SP, Lata J, Chen C, Mai J, Guo F, Tian Z, Ren L, Mao Z, Huang PH, Li P (2018). Digital acoustofluidics enables contactless and programmable liquid handling. Nat Commun.

[99] Chen B, Wu Y, Ao Z, Cai H, Nunez A, Liu Y, Foley J, Nephew K, Lu X, Guo F (2019). High-throughput acoustofluidic fabrication of tumor spheroids. Lab Chip.

[100] Chen K, Sui C, Wu Y, Ao Z, Guo SS, Guo F (2019). A digital acoustofluidic device for on-demand and oil-free droplet generation. Nanotechnology.

[101] Wu Z, Cai H, Ao Z, Nunez A, Liu H, Bondesson M, Guo S, Guo F (2019). A digital acoustofluidic pump powered by localized fluid-substrate interactions. Anal Chem.

[102] Ao Z, Cai H, Wu Z, Johnathon J, Wang H, Mackie K, Guo FJB (2020). Controllable fusion of human brain organoids using acoustofluidics. Lab Chip.

[103] Cai H, Ao Z, Hu L, Moon Y, Wu Z, Lu HC, Kim J, Guo F (2020). Acoustofluidic assembly of 3D neurospheroids to model Alzheimer's disease. Analyst.

[104] Cai H, Ao Z, Wu Z, Nunez A, Jiang L, Carpenter RL, Nephew KP, Guo F (2020). Profiling cell-matrix adhesion using digitalized acoustic streaming. Anal Chem.

[105] Cai H, Wu Z, Ao Z, Nunez A, Chen B, Jiang L, Bondesson M, Guo F (2020). Trapping cell spheroids and organoids using digital acoustofluidics. Biofabrication.

[106] Cai HW, Ao Z, Moon Y, Wu ZH, Lu HC, Kim J, Guo F, Hu LY (2020). Acoustofluidic assembly of 3D neurospheroids to model Alzheimer's disease. Analyst.

[107] Ao Z, Cai H, Wu Z, Ott J, Wang H, Mackie K, Guo F (2021). Controllable fusion of human brain organoids using acoustofluidics. Lab Chip.

[108] Cai H, Ao Z, Wu Z, Song S, Mackie K, Guo F (2021). Intelligent acoustofluidics enabled mini-bioreactors for human brain organoids. Lab Chip.

[109] Ao Z, Wu Z, Cai H, Hu L, Li X, Kaurich C, Chang J, Gu M, Cheng L, Lu X, Guo F (2022). Rapid profiling of tumor-immune interaction using acoustically assembled patient-derived cell clusters. Adv Sci (Weinh).

[110] Zeng Q, Guo F, Yao L, Zhu HW, Zheng L, Guo ZX, Liu W, Chen Y, Guo SS, Zhao XZ (2011). Milliseconds mixing in microfluidic channel using focused surface acoustic wave. Sens Actuators B-Chem.

[111] Chen Y, Ding X, Lin S-CS, Yang S, Huang P-H, Nama N, Zhao Y, Nawaz AA, Guo F, Wang W (2013). Tunable nanowire patterning using standing surface acoustic waves. Acs Nano.

[112] Li S, Ding X, Guo F, Chen Y, Lapsley MI, Lin S-CS, Wang L, McCoy JP, Cameron CE, Huang TJ (2013). An on-chip, multichannel droplet sorter using standing surface acoustic waves. Analyt Chem.

[113] Xie Y, Zhao C, Zhao Y, Li S, Rufo J, Yang S, Guo F, Huang TJ (2013). Optoacoustic tweezers: a programmable, localized cell concentrator based on opto-thermally generated, acoustically activated, surface bubbles. Lab Chip.

[114] Li S, Guo F, Chen Y, Ding X, Li P, Wang L, Cameron CE, Huang TJ (2014). Standing surface acoustic wave based cell coculture. Anal Chem.

[115] Zhao C, Xie Y, Mao Z, Zhao Y, Rufo J, Yang S, Guo F, Mai JD, Huang TJ (2014). Theory and experiment on particle trapping and manipulation via optothermally generated bubbles. Lab Chip.

[116] Guo F, Li P, French JB, Mao Z, Zhao H, Li S, Nama N, Fick JR, Benkovic SJ, Huang TJ (2015). Controlling cell-cell interactions using surface acoustic waves. Proc Natl Acad Sci USA.

[117] Guo F, Xie Y, Li S, Lata J, Ren L, Mao Z, Ren B, Wu M, Ozcelik A, Huang TJ (2015). Reusable acoustic tweezers for disposable devices. Lab Chip.

[118] Guo F, Zhou W, Li P, Mao Z, Yennawar NH, French JB, Huang TJ (2015). Precise manipulation and patterning of protein crystals for macromolecular crystallography using surface acoustic waves. Small.

[119] Li S, Ding X, Mao Z, Chen Y, Nama N, Guo F, Li P, Wang L, Cameron CE, Huang TJ (2015). Standing surface acoustic wave (SSAW)-based cell washing. Lab Chip.

[120] Ren L, Chen Y, Li P, Mao Z, Huang PH, Rufo J, Guo F, Wang L, McCoy JP, Levine SJ, Huang TJ (2015). A high-throughput acoustic cell sorter. Lab Chip.

[121] Chen K, Wu M, Guo F, Li P, Chan CY, Mao Z, Li S, Ren L, Zhang R, Huang TJ (2016). Rapid formation of size-controllable multicellular spheroids via 3D acoustic tweezers. Lab Chip.

[122] Guo F, Mao Z, Chen Y, Xie Z, Lata JP, Li P, Ren L, Liu J, Yang J, Dao M (2016). Three-dimensional manipulation of single cells using surface acoustic waves. Proc Natl Acad Sci.

[123] Lata JP, Guo F, Guo J, Huang PH, Yang J, Huang TJ (2016). Surface acoustic waves grant superior spatial control of cells embedded in hydrogel fibers. Adv Mater.

[124] Mao Z, Xie Y, Guo F, Ren L, Huang PH, Chen Y, Rufo J, Costanzo F, Huang TJ (2016). Experimental and numerical studies on standing surface acoustic wave microfluidics. Lab Chip.

[125] Liu HQ, Ao Z, Cai B, Shu X, Chen KK, Rao L, Luo CL, Wang FB, Liu W, Bondesson M (2018). Size-amplified acoustofluidic separation of circulating tumor cells with removable microbeads. Nano Futures.

[126] Xie Y, Rufo J, Zhong R, Rich J, Li P, Leong KW, Huang TJ (2020). Microfluidic isolation and enrichment of nanoparticles. ACS Nano.

[127] Xie Y, Mao Z, Bachman H, Li P, Zhang P, Ren L, Wu M, Huang TJ (2020). Acoustic cell separation based on density and mechanical properties. J Biomech Eng.

[128] Li P, Mao Z, Peng Z, Zhou L, Chen Y, Huang PH, Truica CI, Drabick JJ, El-Deiry WS, Dao M (2015). Acoustic separation of circulating tumor cells. Proc Natl Acad Sci U S A.

[129] Ding X, Peng Z, Lin SC, Geri M, Li S, Li P, Chen Y, Dao M, Suresh S, Huang TJ (2014). Cell separation using tilted-angle standing surface acoustic waves. Proc Natl Acad Sci U S A.

[130] Wang C, Ma Y, Pei Z, Song F, Zhong J, Wang Y, Yan X, Dai P, Jiang Y, Qiu J (2022). Sheathless acoustic based flow cell sorter for enrichment of rare cells. Cytometry A.

[131] Guo F, Ji X-H, Liu K, He R-X, Zhao L-B, Guo Z-X, Liu W, Guo S-S, Zhao X-Z (2010). Droplet electric separator microfluidic device for cell sorting. Appl Phys Lett.

[132] Guo F, Liu K, Ji X-H, Ding H-J, Zhang M, Zeng Q, Liu W, Guo S-S, Zhao X-Z (2010). Valve-based microfluidic device for droplet on-demand operation and static assay. Appl Phys Lett.

[133] Liu K, Wang H, Chen K-J, Guo F, Lin W-Y, Chen Y-C, Phung DL, Tseng H-R, Shen CK-F (2010). A digital microfluidic droplet generator produces self-assembled supramolecular nanoparticles for targeted cell imaging. Nanotechnology.

[134] Wang H, Liu K, Chen K-J, Lu Y, Wang S, Lin W-Y, Guo F, Kamei K-i, Chen Y-C, Ohashi M (2010). A rapid pathway toward a superb gene delivery system: programming structural and functional diversity into a supramolecular nanoparticle library. Acs Nano.

[135] Ji X-H, Cheng W, Guo F, Liu W, Guo S-S, He Z-K, Zhao X-Z (2011). On-demand preparation of quantum dot-encoded microparticles using a droplet microfluidic system. Lab Chip.

[136] Ji X-H, Zhang N-G, Cheng W, Guo F, Liu W, Guo S-S, He Z-K, Zhao X-Z (2011). Integrated parallel microfluidic device for simultaneous preparation of multiplex optical-encoded microbeads with distinct quantum dot barcodes. J Mater Chem.

[137] Luo T, Fan L, Zhu R, Sun D (2019). Microfluidic single-cell manipulation and analysis: methods and applications. Micromachines (Basel).

[138] Liu K, Lepin EJ, Wang M-W, Guo F, Lin W-Y, Chen Y-C, Sirk SJ, Olma S, Phelps ME, Zhao X-Z (2011). Microfluidic-based (18)F-labeling of biomolecules for immuno-positron emission tomography. Mol Imaging.

[139] Cai B, Guo F, Zhao L, He R, Chen B, He Z, Yu X, Guo S, Xiong B, Liu W, Zhao X (2014). Disk-like hydrogel bead-based immunofluorescence staining toward identification and observation of circulating tumor cells. Microfluid Nanofluid.

[140] Guo F, Lapsley MI, Nawaz AA, Zhao Y, Lin S-CS, Chen Y, Yang S, Zhao X-Z, Huang TJ (2012). A droplet-based, optofluidic device for high-throughput quantitative bioanalysis. Analyt Chem.

[141] Wu Z, Gong Z, Ao Z, Xu J, Cai H, Muhsen M, Heaps S, Bondesson M, Guo S, Guo F (2020). Rapid microfluidic formation of uniform patient-derived breast tumor spheroids. ACS Appl Bio Mater.

[142] Yang S, Guo F, Kiraly B, Mao X, Lu M, Leong KW, Huang TJ (2012). Microfluidic synthesis of multifunctional Janus particles for biomedical applications. Lab Chip.

[143] Farahinia A, Zhang WJ, Badea I (2021). Novel microfluidic approaches to circulating tumor cell separation and sorting of blood cells: a review. J Sci Adv Mater Devices.

[144] Sun Y, Cai B, Wei X, Wang Z, Rao L, Meng QF, Liao Q, Liu W, Guo S, Zhao X (2019). A valve-based microfluidic device for on-chip single cell treatments. Electrophoresis.

[145] Kim H, Kim J (2014). A microfluidic-based dynamic microarray system with single-layer pneumatic valves for immobilization and selective retrieval of single microbeads. Microfluid Nanofluid.

[146] Zhao L, Ma C, Shen S, Tian C, Xu J, Tu Q, Li T, Wang Y, Wang J (2016). Pneumatic microfluidics-based multiplex single-cell array. Biosens Bioelectron.

[147] Zheng L, Wang B, Sun Y, Dai B, Fu Y, Zhang Y, Wang Y, Yang Z, Sun Z, Zhuang S, Zhang D (2021). An oxygen-concentration-controllable multiorgan microfluidic platform for studying hypoxia-induced lung cancer-liver metastasis and screening drugs. ACS Sensors.

[148] Thurgood P, Chheang C, Needham S, Pirogova E, Peter K, Baratchi S, Khoshmanesh K (2022). Generation of dynamic vortices in a microfluidic system incorporating stenosis barrier by tube oscillation. Lab Chip.

[149] Amini H, Lee W, Di Carlo D (2014). Inertial microfluidic physics. Lab Chip.

[150] He Z, Guo F, Feng C, Cai B, Lata JP, He R, Huang Q, Yu X, Rao L, Liu H (2016). Fetal nucleated red blood cell analysis for non-invasive prenatal diagnostics using a nanostructure microchip. J Mater Chem B.

[151] Zhang H, Yang Y, Li X, Shi Y, Hu B, An Y, Zhu Z, Hong G, Yang CJ (2018). Frequency-enhanced transferrin receptor antibody-labelled microfluidic chip (FETAL-Chip) enables efficient enrichment of circulating nucleated red blood cells for non-invasive prenatal diagnosis. Lab Chip.

[152] Wei X, Cai B, Chen K, Cheng L, Zhu Y, Wang Z, Sun Y, Liu W, Guo S, Zhang Y, Zhao X (2019). Enhanced isolation and release of fetal nucleated red blood cells using multifunctional nanoparticle-based microfluidic device for non-invasive prenatal diagnostics. Sensor Actuat B-Chem.

[153] Xu S, Wu L, Qin Y, Jiang Y, Sun K, Holcomb C, Gravett M, Vojtech L, Schiro P, Chiu D (2021). In situsequential ensemble-decision aliquot ranking isolation and fluorescence hybridization identification of rare cells from blood by using concentrated peripheral blood mononuclear cells. Anal Chem.

[154] Sun Y, Li NQ, Cai B, Wei XY, Wang ZX, Cui H, Zhao DS, Zhang YZ, Zhao XZ (2020). A biocompatible nanofibers-based microchip for isolation and nondestructive release of fetal nucleated red blood cells. Adv Mater Interfaces.

[155] Dong J, Chen JF, Smalley M, Zhao M, Ke Z, Zhu Y, Tseng HR (2020). Nanostructured substrates for detection and characterization of circulating rare cells: from materials research to clinical applications. Adv Mater.

[156] Hu X, Zang X, Lv Y (2021). Detection of circulating tumor cells: advances and critical concerns. Oncol Lett.

[157] Chiou A, Hinckley J, Khaitan R, Varsano N, Wang J, Malarkey H, Hernandez C, Williams R, Estroff L, Weiner S (2021). Fluorescent silica nanoparticles to label metastatic tumor cells in mineralized bone microenvironments. Small.

[158] van der Vlist EJ, Nolte-'t Hoen EN, Stoorvogel W, Arkesteijn GJ, Wauben MH (2012). Fluorescent labeling of nano-sized vesicles released by cells and subsequent quantitative and qualitative analysis by high-resolution flow cytometry. Nat Protoc.

[159] Kurdekar AD, Avinash Chunduri LA, Manohar CS, Haleyurgirisetty MK, Hewlett IK, Venkataramaniah K (2018). Streptavidin-conjugated gold nanoclusters as ultrasensitive fluorescent sensors for early diagnosis of HIV infection. Sci Adv.

[160] Li X, Soler M, Szydzik C, Khoshmanesh K, Schmidt J, Coukos G, Mitchell A, Altug H (2018). Label-free optofluidic nanobiosensor enables real-time analysis of single-cell cytokine secretion. Small.

[161] Huang LL, Nie W, Zhang J, Xie HY (2020). Cell-membrane-based biomimetic systems with bioorthogonal functionalities. Acc Chem Res.

[162] Chen M, Cui Y, Hao W, Fan Y, Zhang J, Liu Q, Jiang M, Yang Y, Wang Y, Gao C (2021). Ligand-modified homologous targeted cancer cell membrane biomimetic nanostructured lipid carriers for glioma therapy. Drug Deliv.

[163] Liu H, Li Y, Sun K, Fan J, Zhang P, Meng J, Wang S, Jiang L (2013). Dual-responsive surfaces modified with phenylboronic acid-containing polymer brush to reversibly capture and release cancer cells. J Am Chem Soc.

[164] Li J, Qi C, Lian Z, Han Q, Wang X, Cai S, Yang R, Wang C (2016). Cell-capture and release platform based on peptide-aptamer-modified nanowires. ACS Appl Mater Inter.

[165] Abdolahad M, Taghinejad M, Taghinejad H, Janmaleki M, Mohajerzadeh S (2012). A vertically aligned carbon nanotube-based impedance sensing biosensor for rapid and high sensitive detection of cancer cells. Lab Chip.

[166] Jeon S, Hong W, Lee ES, Cho Y (2014). High-purity isolation and recovery of circulating tumor cells using conducting polymer-deposited microfluidic device. Theranostics.

[167] Shen H, Su R, Peng J, Zhu L, Deng K, Niu Q, Song Y, Yang L, Wu L, Zhu Z, Yang C (2022). Antibody-engineered red blood cell interface for high-performance capture and release of circulating tumor cells. Bioactive Mater.

[168] Li R, He Y, Zhang S, Qin J, Wang J (2018). Cell membrane-based nanoparticles: a new biomimetic platform for tumor diagnosis and treatment. Acta Pharm Sin B.

[169] Chang ZM, Zhou H, Yang C, Zhang R, You Q, Yan R, Li L, Ge M, Tang Y, Dong WF, Wang Z (2020). Biomimetic immunomagnetic gold hybrid nanoparticles coupled with inductively coupled plasma mass spectrometry for the detection of circulating tumor cells. J Mater Chem B.

[170] Wang Z, Cheng L, Sun Y, Wei X, Cai B, Liao L, Zhang Y, Zhao XZ (2021). Enhanced isolation of fetal nucleated red blood cells by enythrocyte-leukocyte hybrid membrane-coated magnetic nanoparticles for noninvasive pregnant diagnostics. Anal Chem.

[171] Wang S, Wang H, Jiao J, Chen KJ, Owens GE, Kamei K, Sun J, Sherman DJ, Behrenbruch CP, Wu H, Tseng HR (2009). Three-dimensional nanostructured substrates toward efficient capture of circulating tumor cells. Angew Chem Int Ed Engl.

[172] Sun N, Li X, Wang Z, Zhang R, Wang J, Wang K, Pei R (2016). A Multiscale TiO2 nanorod array for ultrasensitive capture of circulating tumor cells. ACS Appl Mater Inter.

[173] Ma GC, Lin WH, Huang CE, Chang TY, Liu JY, Yang YJ, Lee MH, Wu WJ, Chang YS, Chen M (2019). A Silicon-based coral-like nanostructured microfluidics to isolate rare cells in human circulation: validation by SK-BR-3 cancer cell line and its utility in circulating fetal nucleated red blood cells. Micromachines (Basel).

[174] Qiu JC, Zhao K, Li LL, Yu X, Guo WB, Wang S, Zhang XD, Pan CF, Wang ZL, Liu H (2017). A titanium dioxide nanorod array as a high-affinity nano-bio interface of a microfluidic device for efficient capture of circulating tumor cells. Nano Res.

[175] Zhang P, Chen L, Xu T, Liu H, Liu X, Meng J, Yang G, Jiang L, Wang S (2013). Programmable fractal nanostructured interfaces for specific recognition and electrochemical release of cancer cells. Adv Mater.

[176] Dou X, Li P, Jiang S, Bayat H, Schönherr H (2017). Bioinspired hierarchically structured surfaces for efficient capture and release of circulating tumor cells. ACS Appl Mater Inter.

[177] Ding P, Wang Z, Wu Z, Zhou Y, Sun N, Pei R (2020). Natural biointerface based on cancer cell membranes for specific capture and release of circulating tumor cells. ACS Appl Mater Inter.

[178] Zhao Y, Li A, Jiang L, Gu Y, Liu J (2021). Hybrid membrane-coated biomimetic nanoparticles (HM@BNPs): a multifunctional nanomaterial for biomedical applications. Biomacromol.

[179] Parker SG, Yang Y, Ciampi S, Gupta B, Kimpton K, Mansfeld FM, Kavallaris M, Gaus K, Gooding JJ (2018). A photoelectrochemical platform for the capture and release of rare single cells. Nat Commun.

[180] Timilsena S, Ardsiri S, Lerdwana S, Manandhar KD, Pattanapanyasat K, Noulsri E (2022). Accuracy of lymphocyte counts from UniCel DxH 800 in beta-thalassemia/HbE patients having various numbers of nucleated red blood cells. Asian Pac J Allergy Immunol.

[181] Zhu W, Zhang XY, Marjani SL, Zhang J, Zhang W, Wu S, Pan X (2017). Next-generation molecular diagnosis: single-cell sequencing from bench to bedside. Cell Mol Life Sci.

[182] Schuring-Blom GH, Hoovers JM, van Lith JM, Knegt AC, Leschot NJ (2001). FISH analysis of fetal nucleated red blood cells from CVS washings in cases of aneuploidy. Prenat Diagn.

[183] Zhen DK, Wang JY, Falco VM, Weber W, Delli-Bovi L, Bianchi DW (1998). Poly-FISH: a technique of repeated hybridizations that improves cytogenetic analysis of fetal cells in maternal blood. Prenat Diagn.

[184] Butler JM (2003). Recent developments in Y-short Tandem repeat and Y-single nucleotide polymorphism analysis. Forensic Sci Rev.

[185] Holland C, Tanevski J, Perales-Patón J, Gleixner J, Kumar M, Mereu E, Joughin B, Stegle O, Lauffenburger D, Heyn H (2020). Robustness and applicability of transcription factor and pathway analysis tools on single-cell RNA-seq data. Genome Biol.

[186] Dekairelle AF, Hoste B (2001). Application of a Y-STR-pentaplex PCR (DYS19, DYS389I and II, DYS390 and DYS393) to sexual assault cases - ScienceDirect. Forensic Sci Int.

[187] Osamu S, Satoshi S, Johnson KL, Barbara P, Steven R, Delli-Bovi LC, Bianchi DW (2001). Diagnosis of trisomy 21 in fetal nucleated erythrocytes from maternal blood by use of short tandem repeat sequences. Clin Chem.

[188] Giambona A, Leto F, Damiani G, Jakil C, Cigna V, Schillaci G, Stampone G, Volpes A, Allegra A, Nicolaides KH (2016). Identification of embryo-fetal cells in celomic fluid using morphological and short-tandem repeats analysis. Prenat Diagn.

[189] Pertl B, Yau SC, Sherlock J, Davies AF, Mathew CG, Adinolfi M (1994). Rapid molecular method for prenatal detection of Down's syndrome. Lancet.

[190] Yoon HR, Park YS, Kim YK (2002). Rapid prenatal detection of down and Edwards syndromes by fluorescent polymerase chain reaction with short tandem repeat markers. Yonsei Med J.

[191] Mann K, Petek E, Pertl B, Levy B (2019). Prenatal detection of chromosome aneuploidy by quantitative fluorescence PCR. Prenat Diagn.

[192] Parks M, Court S, Bowns B, Cleary S, Clokie S, Hewitt J, Williams D, Cole T, MacDonald F, Griffiths M, Allen S (2017). Non-invasive prenatal diagnosis of spinal muscular atrophy by relative haplotype dosage. Eur J Hum Genet.

[193] Chamberlain JS, Chamberlain JR, Fenwick RG, Ward PA, Caskey CT, Dimnik LS, Bech-Hansen NT, Hoar DI, Richards S, Covone AE (1992). Diagnosis of Duchenne and Becker muscular dystrophies by polymerase chain reaction A multicenter study. JAMA.

[194] Pertl B, Weitgasser U, Kopp S, Kroisel PM, Sherlock J, Adinolfi M (1996). Rapid detection of trisomies 21 and 18 and sexing by quantitative fluorescent multiplex PCR. Hum Genet.

[195] Bryndorf T, Kirchhoff M, Rose H, Maahr J, Gerdes T, Karhu R, Kallioniemi A, Christensen B, Lundsteen C, Philip J (1995). Comparative genomic hybridization in clinical cytogenetics. Am J Hum Genet.

[196] Kallioniemi A, Kallioniemi OP, Sudar D, Rutovitz D, Gray JW, Waldman F, Pinkel D (1992). Comparative genomic hybridization for molecular cytogenetic analysis of solid tumors. Science.

[197] Levy B, Dunn TM, Kaffe S, Kardon N, Hirschhorn K (1998). Clinical applications of comparative genomic hybridization. Genet Med.

[198] Le Caignec C, Boceno M, Saugier-Veber P, Jacquemont S, Joubert M, David A, Frebourg T, Rival JM (2005). Detection of genomic imbalances by array based comparative genomic hybridisation in fetuses with multiple malformations. J Med Genet.

[199] Cheung SW, Shaw CA, Scott DA, Patel A, Sahoo T, Bacino CA, Pursley A, Li J, Erickson R, Gropman AL (2007). Microarray-based CGH detects chromosomal mosaicism not revealed by conventional cytogenetics. Am J Med Genet A.

[200] Simovich MJ, Yatsenko SA, Kang SH, Cheung SW, Dudek ME, Pursley A, Ward PA, Patel A, Lupski JR (2007). Prenatal diagnosis of a 9q34.3 microdeletion by array-CGH in a fetus with an apparently balanced translocation. Prenat Diagn.

[201] Ballif BC, Kashork CD, Saleki R, Rorem E, Sundin K, Bejjani BA, Shaffer LG (2006). Detecting sex chromosome anomalies and common triploidies in products of conception by array-based comparative genomic hybridization. Prenat Diagn.

[202] Tyreman M, Abbott KM, Willatt LR, Nash R, Lees C, Whittaker J, Simonic I (2009). High resolution array analysis: diagnosing pregnancies with abnormal ultrasound findings. J Med Genet.

[203] Brunetti-Pierri N, Berg JS, Scaglia F, Belmont J, Bacino CA, Sahoo T, Lalani SR, Graham B, Lee B, Shinawi M (2008). Recurrent reciprocal 1q21.1 deletions and duplications associated with microcephaly or macrocephaly and developmental and behavioral abnormalities. Nat Genet.

[204] Shuster E (2007). Microarray genetic screening: a prenatal roadblock for life?. Lancet.

[205] Srebniak M, Boter M, Oudesluijs G, Joosten M, Govaerts L, Van Opstal D, Galjaard RJ (2011). Application of SNP array for rapid prenatal diagnosis: implementation, genetic counselling and diagnostic flow. Eur J Hum Genet.

[206] Srivastava P (2016). Next generation sequencing facilitates disease discoveries. Genetic Clin.

[207] Mardis ER (2008). Next-generation DNA sequencing methods. Annu Rev Genomics Hum Genet.

[208] Wang Z, Gerstein M, Snyder M (2009). RNA-Seq: a revolutionary tool for transcriptomics. Nat Rev Genet.

[209] Mavrou A, Kouvidi E, Antsaklis A, Souka A, Kitsiou Tzeli S, Kolialexi A (2007). Identification of nucleated red blood cells in maternal circulation: a second step in screening for fetal aneuploidies and pregnancy complications. Prenat Diagn.

[210] Chan KC, Jiang P, Sun K, Cheng YK, Tong YK, Cheng SH, Wong AI, Hudecova I, Leung TY, Chiu RW, Lo YM (2016). Second generation noninvasive fetal genome analysis reveals de novo mutations, single-base parental inheritance, and preferred DNA ends. Proc Natl Acad Sci U S A.

[211] Papasavva T, van Ijcken WF, Kockx CE, van den Hout MC, Kountouris P, Kythreotis L, Kalogirou E, Grosveld FG, Kleanthous M (2013). Next generation sequencing of SNPs for non-invasive prenatal diagnosis: challenges and feasibility as illustrated by an application to beta-thalassaemia. Eur J Hum Genet.

[212] Hua R, Barrett AN, Tan TZ, Huang Z, Mahyuddin AP, Ponnusamy S, Sandhu JS, Ho SS, Chan JK, Chong S (2015). Detection of aneuploidy from single fetal nucleated red blood cells using whole genome sequencing. Prenat Diagn.

[213] Vermeesch JR, Voet T, Devriendt K (2016). Prenatal and pre-implantation genetic diagnosis. Nat Rev Genet.

[214] Yin X, Tan K, Vajta G, Jiang H, Tan Y, Zhang C, Chen F, Chen S, Zhang C, Pan X (2013). Massively parallel sequencing for chromosomal abnormality testing in trophectoderm cells of human blastocysts. Biol Reprod.

[215] Lo YM, Corbetta N, Chamberlain PF, Rai V, Sargent IL, Redman CW, Wainscoat JS (1997). Presence of fetal DNA in maternal plasma and serum. Lancet.

[216] Talkowski ME, Ernst C, Heilbut A, Chiang C, Hanscom C, Lindgren A, Kirby A, Liu S, Muddukrishna B, Ohsumi TK (2011). Next-generation sequencing strategies enable routine detection of balanced chromosome rearrangements for clinical diagnostics and genetic research. Am J Hum Genet.

[217] Cirulli ET, Goldstein DB (2010). Uncovering the roles of rare variants in common disease through whole-genome sequencing. Nat Rev Genet.

[218] Dewey FE, Grove ME, Pan C, Goldstein BA, Bernstein JA, Chaib H, Merker JD, Goldfeder RL, Enns GM, David SP (2014). Clinical interpretation and implications of whole-genome sequencing. JAMA.

[219] Lee W, Jiang Z, Liu J, Haverty PM, Guan Y, Stinson J, Yue P, Zhang Y, Pant KP, Bhatt D (2010). The mutation spectrum revealed by paired genome sequences from a lung cancer patient. Nature.

[220] Lau TK, Jiang FM, Stevenson RJ, Lo TK, Chan LW, Chan MK, Lo PSS, Wang W, Zhang HY, Chen F, Choy KW (2013). Secondary findings from non-invasive prenatal testing for common fetal aneuploidies by whole genome sequencing as a clinical service. Prenat Diagn.

[221] Diagnosis. ISfP, Medicine. TSfMaF, Foundation TPQ (2018). Joint Position Statement from the International Society for Prenatal Diagnosis (ISPD), the Society for Maternal Fetal Medicine (SMFM), and the Perinatal Quality Foundation (PQF) on the use of genome-wide sequencing for fetal diagnosis. Prenat Diagn..

[222] Chen F, Liu P, Gu Y, Zhu Z, Nanisetti A, Lan Z, Huang Z, Liu JS, Kang X, Deng Y (2017). Isolation and whole genome sequencing of fetal cells from maternal blood towards the ultimate non-invasive prenatal testing. Prenat Diagn.

[223] Johnston JJ, Lewis KL, Ng D, Singh LN, Wynter J, Brewer C, Brooks BP, Brownell I, Candotti F, Gonsalves SG (2015). Individualized iterative phenotyping for genome-wide analysis of loss-of-function mutations. Am J Hum Genet.

[224] Best S, Wou K, Vora N, Van der Veyver IB, Wapner R, Chitty LS (2018). Promises, pitfalls and practicalities of prenatal whole exome sequencing. Prenat Diagn.

[225] Rabbani B, Tekin M, Mahdieh N (2014). The promise of whole-exome sequencing in medical genetics. J Hum Genet.

[226] Belkadi A, Bolze A, Itan Y, Cobat A, Vincent QB, Antipenko A, Shang L, Boisson B, Casanova JL, Abel L (2015). Whole-genome sequencing is more powerful than whole-exome sequencing for detecting exome variants. P Natl Acad Sci USA.

