# Correction: Noninvasive prenatal diagnosis targeting fetal nucleated red blood cells

**DOI:** 10.1186/s12951-023-01805-6

**Published:** 2023-02-27

**Authors:** Yanyu Chen, Zhuhao Wu, Joseph Sutlive, Ke Wu, Lu Mao, Jiabao Nie, Xing-Zhong Zhao, Feng Guo, Zi Chen, Qinqin Huang

**Affiliations:** 1 The Research and Application Center of Precision Medicine, The Second Affiliated Hospital of Zhengzhou University, Zhengzhou University, Zhengzhou, 450052 China; 2 School of Physics and Technology, Wuhan University, Wuhan, 430072 China; 3 Department of Intelligent Systems Engineering, Indiana University, Bloomington, IN 47405 USA; 4 Division of Thoracic and Cardiac Surgery, Brigham and Women’s Hospital and Harvard Medical School, Boston, MA 02115 USA; 5 Department of Biological Sciences, Northeastern University, Boston, MA 02115 USA

**Correction: Journal of Nanobiotechnology (2022) 20:546** **https://doi.org/10.1186/s12951-022-01749-3**

Following publication of the original article [1], the authors would like to correct the affiliations of all the authors.

The revised affiliations are provided below.

1. The Research and Application Center of Precision Medicine, The Second Affiliated Hospital of Zhengzhou University, Zhengzhou University, Zhengzhou 450052, China
2. School of Physics and Technology, Wuhan University, Wuhan 430072, China
3. Department of Intelligent Systems Engineering, Indiana University, Bloomington, IN 47405, USA
4. Division of Thoracic and Cardiac Surgery, Brigham and Women's Hospital and Harvard Medical School, Boston, MA 02115, USA
5. Department of Biological Sciences, Northeastern University, Boston, MA 02115, USA

The original article has been corrected.
